# NanoE-Tox: New and in-depth database concerning ecotoxicity of nanomaterials

**DOI:** 10.3762/bjnano.6.183

**Published:** 2015-08-25

**Authors:** Katre Juganson, Angela Ivask, Irina Blinova, Monika Mortimer, Anne Kahru

**Affiliations:** 1 Laboratory of Environmental Toxicology, National Institute of Chemical Physics and Biophysics, Akadeemia tee 23, 12618 Tallinn, Estonia; 2 Department of Chemistry, Tallinn University of Technology, Akadeemia tee 15, 12618 Tallinn, Estonia; 3 Mawson Institute, University of South Australia, Mawson Lakes, 5095 South Australia, Australia; 4 Bren School of Environmental Science & Management, University of California Santa Barbara, Santa Barbara, California 93106-5131, United States

**Keywords:** nanoparticles, physico-chemical properties, REACH, Thomson Reuters Web of Science, toxicity mechanisms

## Abstract

The increasing production and use of engineered nanomaterials (ENMs) inevitably results in their higher concentrations in the environment. This may lead to undesirable environmental effects and thus warrants risk assessment. The ecotoxicity testing of a wide variety of ENMs rapidly evolving in the market is costly but also ethically questionable when bioassays with vertebrates are conducted. Therefore, alternative methods, e.g., models for predicting toxicity mechanisms of ENMs based on their physico-chemical properties (e.g., quantitative (nano)structure-activity relationships, QSARs/QNARs), should be developed. While the development of such models relies on good-quality experimental toxicity data, most of the available data in the literature even for the same test species are highly variable. In order to map and analyse the state of the art of the existing nanoecotoxicological information suitable for QNARs, we created a database NanoE-Tox that is available as Supporting Information File 1. The database is based on existing literature on ecotoxicology of eight ENMs with different chemical composition: carbon nanotubes (CNTs), fullerenes, silver (Ag), titanium dioxide (TiO_2_), zinc oxide (ZnO), cerium dioxide (CeO_2_), copper oxide (CuO), and iron oxide (FeO*_x_*; Fe_2_O_3_, Fe_3_O_4_). Altogether, NanoE-Tox database consolidates data from 224 articles and lists altogether 1,518 toxicity values (EC_50_/LC_50_/NOEC) with corresponding test conditions and physico-chemical parameters of the ENMs as well as reported toxicity mechanisms and uptake of ENMs in the organisms. 35% of the data in NanoE-Tox concerns ecotoxicity of Ag NPs, followed by TiO_2_ (22%), CeO_2_ (13%), and ZnO (10%). Most of the data originates from studies with crustaceans (26%), bacteria (17%), fish (13%), and algae (11%). Based on the median toxicity values of the most sensitive organism (data derived from three or more articles) the toxicity order was as follows: Ag > ZnO > CuO > CeO_2_ > CNTs > TiO_2_ > FeO*_x_*. We believe NanoE-Tox database contains valuable information for ENM environmental hazard estimation and development of models for predicting toxic potential of ENMs.

## Introduction

**Figure 1:**
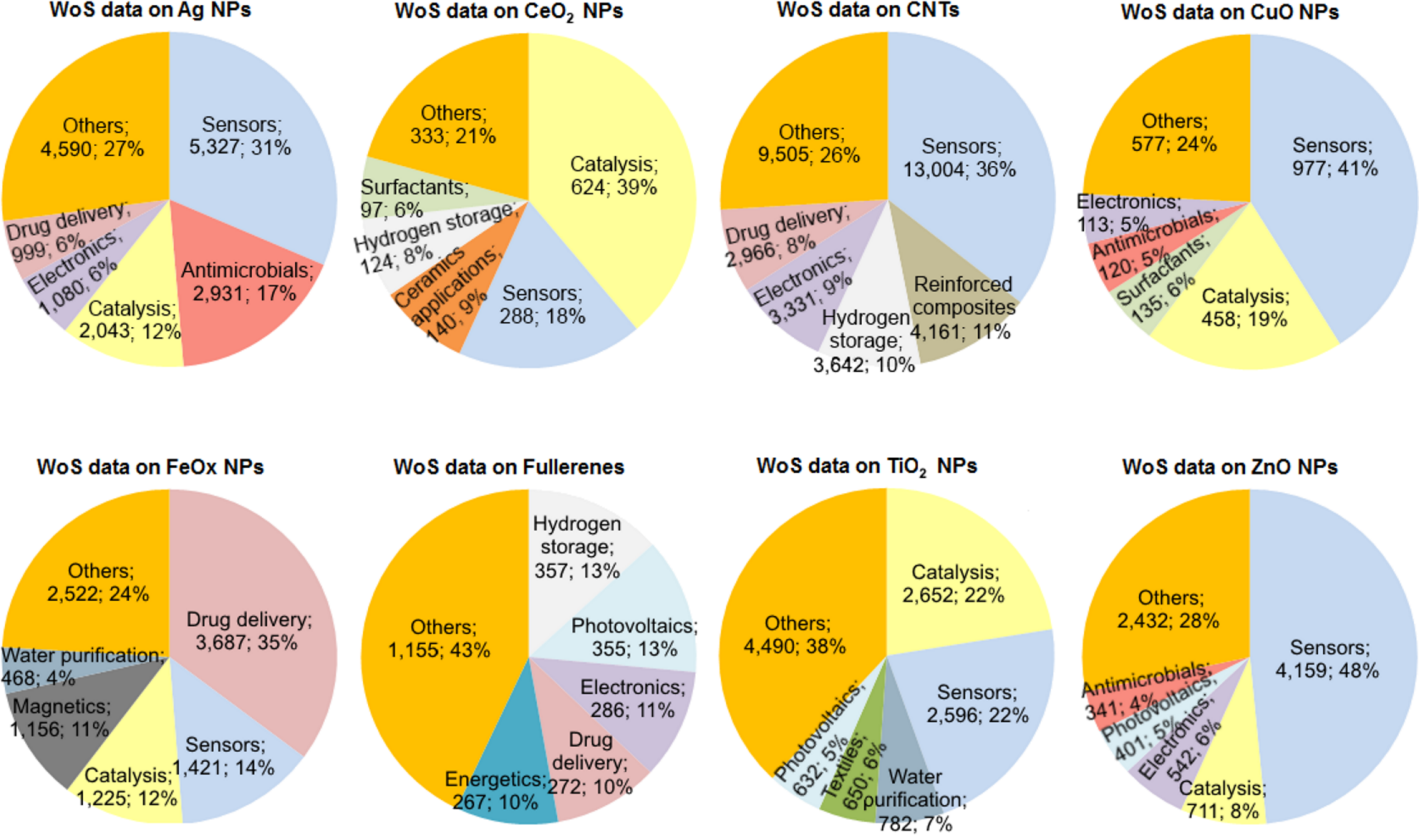
Proposed fields of application of engineered nanomaterials (ENMs) according to the publications in Thomson Reuters WoS. Keywords were selected from the review by Bondarenko et al. [4]. Numbers below each application category indicate the number and share of papers retrieved. The numerical data are presented in Table S1 (Supporting Information File 2). The bibliometric data search was performed in Thomson Reuters WoS on March 19, 2015.

The production and use of engineered nanomaterials (ENMs) in consumer products is increasing rapidly [1]. As of March 20, 2015 there were more than 1,800 products listed in Consumer Products Inventory [2]. According to this inventory, the most abundant ENMs used in consumer products are silver (438 products), titanium (107), carbon (90), silica (81), zinc (38) and gold (24) with the main applications in antimicrobial protection (381 products), coatings (188) and health products (142). The number of published articles could serve as a good indicator of the potential future use of ENMs. A search performed on March 19, 2015 in Thomson Reuters Web of Science (WoS) with the keywords chosen based on Aitken et al. [3] and Bondarenko et al. [4] and listed in Table S1 (Supporting Information File 2) revealed that the majority of the papers concerned the applications of carbon nanotubes (36,609 papers, 40%), followed by Ag nanoparticles (NPs; 16,970, 19%), TiO_2_ NPs (11,802, 13%), and iron oxide NPs (10,479, 11%) while the most common fields of application were sensors (28,027 papers, 31%), catalysis (10,435, 11%) and drug delivery (8,838, 10%) (Figure 1, Table S1, Supporting Information File 2). However, the exact production volumes of ENMs are not publicly available [4]. Piccinno et al. estimated based on a survey sent to companies producing and using ENMs that the most produced ENMs were TiO_2_ (550–5,500 t/year), SiO_2_ (55–55,000 t/year), AlO*_x_* (55–5,500 t/year), ZnO (55–550 t/year), carbon nanotubes (CNT; 55–550 t/year), FeO*_x_* (5.5–5,500 t/year), CeO*_x_* and Ag (both 5.5–550 t/year), fullerenes and quantum dots (both 0.6-5.5 t/year) [5]. Warningly, the increasing production and use of ENMs leads inevitably to their higher concentrations in the environment. Thus, the risks caused by ENMs both to humans and the environment need to be assessed [6].

Risk assessment of all the ENMs in the market would require the sacrifice of enormous amounts of test organisms of diverse range [7]. Therefore, there is a need to refine, reduce or replace (3R’s) animal testing and develop alternative risk evaluation methods [7–8]. Recently, the categorisation of ENMs based on their physico-chemical properties, exposure and use scenarios and biological effects was suggested as a strategy to facilitate regulatory decision making while minimising time-consuming and costly in vivo studies [9]. In addition to high-throughput screening tests, modelling can provide information for rapid assessment of the toxicity mechanisms of ENMs [10]. For instance, models based on dynamic energy budget (DEB) theory have been developed for predicting toxicity mechanisms of ENMs [11]. Also, quantitative (nano)structure-activity relationship (QSARs/QNARs) models have great potential for predicting the harmful effects of ENMs from their physical, chemical, and morphological properties that can be measured experimentally or computed based on the ENMs structure [12]. Development of in silico methods relies on good-quality experimental data on ENM toxicity as the set of parameters which determine the toxic potential of each type of ENMs in specific test species/taxa is largely unknown [13].

In order to relate the toxic effects of ENMs to their physico-chemical properties and reveal the data gaps, the existing data have to be carefully collected and analysed. One increasingly popular approach in systematically collecting and organising available data on nanomaterials is creating databases. In 2012, Hristozov et al. emphasised that the available data on nanomaterials in environmental, health and safety databases and online chemical databases were very scarce [14]. Recently, a databases working group was established in the framework of European Union NanoSafety Cluster [15] which highlights the importance of development of in-depth databases on ENMs. In addition, nanotoxicity-related databases are developed and supported at national level in EU. For instance, in Germany an application-based nanomaterial database, which includes information on potential toxicological effects of ENMs, has been created in the DaNa project [16–17]. In Denmark, a database that focuses on potential risks of ENM containing products, "The Nanodatabase", has been developed [18]. The latter lists currently 1,425 products and introduces NanoRiskCat that evaluates ENMs risk according to potential exposure and hazard potential of these ENMs to humans and environment [19]. However, the risk estimations are derived from the available literature on the effects of nanomaterials but not on the actual risk assessment of the specific ENM-containing products. Therefore, the risk levels reported in the database do not account for concentrations or the physico-chemical properties of the specific ENMs used in the products. Independent online databases containing nanotoxicological information have also been created in other countries outside Europe. For instance, NanoToxdb: A database on Nanomaterial Toxicity [20] that is by description a comprehensive database containing information on nanomaterials toxicity to *Daphnia magna.* However, it contains altogether only 32 EC_50_ values for 10 different ENMs and contains no references for the toxicity data. Moreover, no information on physico-chemical properties of ENMs except primary particle size has been included in the database and regarding testing conditions, only the test duration is reported in a few cases. As a different approach, some databases, e.g., NHECD (Knowledge on the Health, Safety and Environmental Impact of Nanoparticles) [21] and Hazardous Substances Data Bank [22] comprise nanotoxicological papers.

In this communication we present a nanoecotoxicological database based on existing literature data on ecotoxicity of selected ENMs. In addition to quantitative toxicity data (e.g., EC_50_ values) information on physico-chemical properties of ENMs and testing conditions as well as on reported mechanisms and uptake of ENMs in the organisms was compiled. All the collected data were analysed to give an overview of ENM toxicity across different studied species. The following ENMs based on production volumes, application in consumer products and technological potential were included in the database: carbon nanotubes (CNTs), fullerenes, silver (Ag), titanium dioxide (TiO_2_), zinc oxide (ZnO), cerium dioxide (CeO_2_), copper oxide (CuO), and iron oxide (FeO*_x_*; Fe_2_O_3_, Fe_3_O_4_). Furthermore, all these ENMs, except CuO, are listed by the Organisation for Economic Co-operation and Development (OECD) Working Party on Manufactured Nanomaterials as ‘commercially relevant’ representative manufactured nanomaterials to be investigated under the OECD sponsorship programme [23]. We believe the database presented in this paper contains valuable information for ENM environmental hazard estimation and development of models, including valid QSAR models, for predicting toxic potential of ENMs.

## Methodology

The process of creating the nanoecotoxicological database can be roughly divided into three steps: selecting keywords for literature search, performing the literature search in Thomson Reuters WoS, collecting and classification of information from retrieved papers into a database. As the selection of keywords is critical in this type of data collection, all the keywords used in this study are listed in Table 1. To find different possible types of ‘nano’ materials, i.e., nanoparticles, nanomaterials, nanotubes, a truncated search term “nano*” was selected. In order to give equal weight to all ecotoxicological test species, the restricting keyword “ecotoxic*” was used instead of organism-specific keywords. Thus, inevitably some of the ecotoxicological data on ENMs has been unintentionally excluded from the database because not all articles reporting studies on nanotoxicity to environmentally relevant organisms necessarily use terms “ecotoxic”, “ecotoxicity” or “ecotoxicology”. When performing the search, truncated names, molecular formulas and/or common abbreviations of the 8 NPs were used (Table 1).

**Table 1:** Keywords used for bibliometric data search in Thomson Reuters WoS database.

ENM	Keywords

Ag	(nano* AND ecotoxic* AND silver) OR (nano* AND ecotoxic* AND Ag)
CeO_2_	(nano* AND ecotoxic* AND cerium *oxide) OR (nano* AND ecotoxic* AND ceria) OR (nano* AND ecotoxic* AND CeO2)
CNT	(nano* AND ecotoxic* AND carbon nanotu*) OR (nano* AND ecotoxic* AND CNT) OR (nano* AND ecotoxic* AND *CNT)
CuO	(nano* AND ecotoxic* AND copper oxide) OR (nano* AND ecotoxic* AND CuO)
FeO*_x_*	(nano* AND ecotoxic* AND iron *oxide) OR (nano* AND ecotoxic* AND Fe3O4) OR (nano* AND ecotoxic* AND Fe2O3)
fullerene	(nano* AND ecotoxic* AND fulleren*)
TiO_2_	(nano* AND ecotoxic* AND titanium *oxide) OR (nano* AND ecotoxic* AND titania) OR (nano* AND ecotoxic* AND TiO2)
ZnO	(nano* AND ecotoxic* AND zinc oxide) OR (nano* AND ecotoxic* AND ZnO)

Thomson Reuters WoS database – one of the largest international and multidisciplinary databases available, covering the most comprehensive list of journals published in English – was used for the bibliometric data search. Using WoS (all databases, all years) for the keyword searches enabled us to compare the data collected into NanoE-Tox with analyses performed in our previous reviews [4,8,24–25]. The search was performed on a regular basis from October 2012 to January 6, 2015. From each paper that was retrieved using the keywords specified in Table 1, maximum available information on physico-chemical properties of ENMs and the toxicity data were extracted and tabulated. It is important to note that in the earlier papers dating back 10 years from now, the NPs characterisation was often limited to their primary size. In more recent nanotoxicological articles, set of parameters required for characterisation of ENMs generally include chemical composition, purity, primary particle size, shape, surface area, coating, agglomeration and/or aggregation, hydrodynamic size in the aqueous test medium, surface charge, stability and solubility of ENMs. For the current NanoE-Tox database (Supporting Information File 1) we collected the following properties of the pristine NPs: chemical composition, origin (producer/in-house synthesised), shape, coating, primary size (diameter and length if applicable), impurities, surface area, and other reported observations. For the characterisation of ENMs in the test environment the following information was registered: test medium, hydrodynamic size of NPs in the test environment (including the method used for analysis), dissolution (if applicable), and surface charge (ζ-potential). Concerning the toxicity testing, we tabulated the following information: test organism, test medium, test duration, temperature, illumination and other reported conditions, toxicity endpoint/measure (e.g., EC_50_, LC_50_, NOEC), obtained toxicity value, and other reported observations. In addition, each paper was analysed to find information concerning (i) specific mechanism of toxicity of the studied ENM (Table S2, Supporting Information File 2) (ii) uptake in the organisms, and (iii) accumulation in cells, tissues and organs (Table S3, Supporting Information File 2). All the collected data were compiled into a Microsoft Excel spreadsheet which was used for creating a database on ecotoxicology of engineered nanomaterials, NanoE-Tox (Supporting Information File 1).

## Results and Discussion

During the recent years, the number of peer-reviewed papers related to nanoecotoxicology has increased exponentially. According to Thomson Reuters WoS, 770 nanoecotoxicological peer-reviewed papers that corresponded to keywords “nano* AND ecotoxic*” were published between 2006 and March 2015. The rapidly increasing number of scientific publications on ecotoxicity of ENMs over the past decade, has inspired several review articles summarising the existing data in the field [4,8,13,24–31]. However, each review has focused on specific aspects and parameters of ENMs testing; therefore, it is difficult to get an overview of all the factors (and their values) that might influence the toxicity of ENMs. We have previously collected and analysed ecotoxicological data for seven different NPs (TiO_2_, ZnO, CuO, Ag, SWCNTs, MWCNTs and C_60_ fullerenes) and seven organism groups representing different trophic levels (bacteria, algae, crustaceans, ciliates, fish, yeasts and nematodes). Altogether 77 toxicity values were analysed [24]. In our recent review [4], we summarised the recent research on toxicological and ecotoxicological findings for Ag, CuO and ZnO NPs including more than 300 toxicity values. In addition to ecotoxicological test species the toxic effects of studied NPs toward mammalian cells in vitro were reviewed [4]. The bibliographic search performed in the current study by using keywords listed in Table 1 resulted in nearly 500 individual papers. All the papers were thoroughly studied for ecotoxicity data. Unfortunately, many of the retrieved papers either did not concern the NP of interest or were review articles. In addition, the importance of including synonyms in keywords to increase the number of relevant articles in search results was apparent (Table 1). For example, the search using keywords “nano* AND ecotoxic* AND cerium *oxide” resulted in 30 papers, whereas “nano* AND ecotoxic* AND CeO2” resulted in 34 papers; remarkably, only 20 papers overlapped. The latter example was also true for other ENMs.

### Analysis of the database: general overview of the sources and contents of the papers

**Figure 2:**
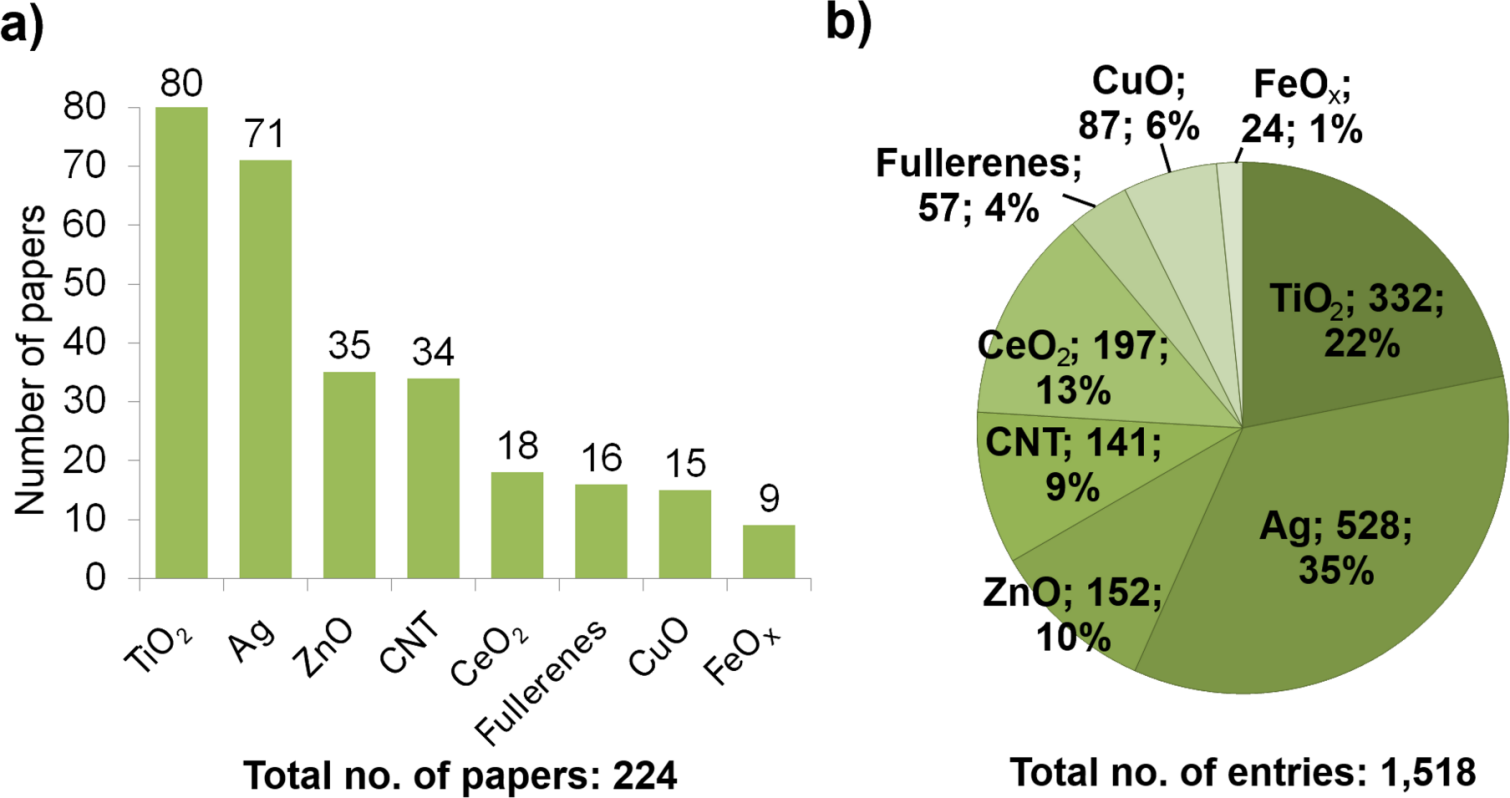
Information in the NanoE-Tox database for different types of engineered nanomaterials (ENMs): (a) number of scientific papers in the database and (b) number and share of entries for each of the tested nanoparticles (ENM; number of entries; share of entries). One entry equals one line that includes all the ENM parameters and toxicity test details. The database entries were selected based on bibliometric data search in Thomson Reuters WoS using the keywords as indicated in Table 1 as of January 6, 2015.

The search in Thomson Reuters WoS using the time span of “all years” indicated that all the papers about ecotoxicity of ENMs have been published within the last ten years. Almost half of the papers retrieved from the initial bibliographic search, 224 of 500 articles from 66 journals, contained relevant nanotoxicological information and were included in NanoE-Tox database (Supporting Information File 1). From these studies 1,518 toxicity values were recorded with test conditions on toxicity testing and physico-chemical parameters of NPs linked to the toxicity data (further designated as ‘database entry’). Out of 224 scientific papers that were selected for the database the largest number of papers concerned TiO_2_ and Ag (80 and 71, respectively) followed by ZnO and CNTs (35 and 34 papers). For CeO_2_, fullerenes and CuO, 15–18 papers were found and the lowest number of papers was retrieved for FeO*_x_* (Figure 2a). From the 1,518 toxicity values (entries) in the database, the highest percentage (35%) concerned Ag followed by TiO_2_ (22%), CeO_2_ (13%), ZnO (10%), CNTs (9%), CuO (6%), fullerenes (4%) and FeO*_x_* (1%) (Figure 2b).

**Figure 3:**
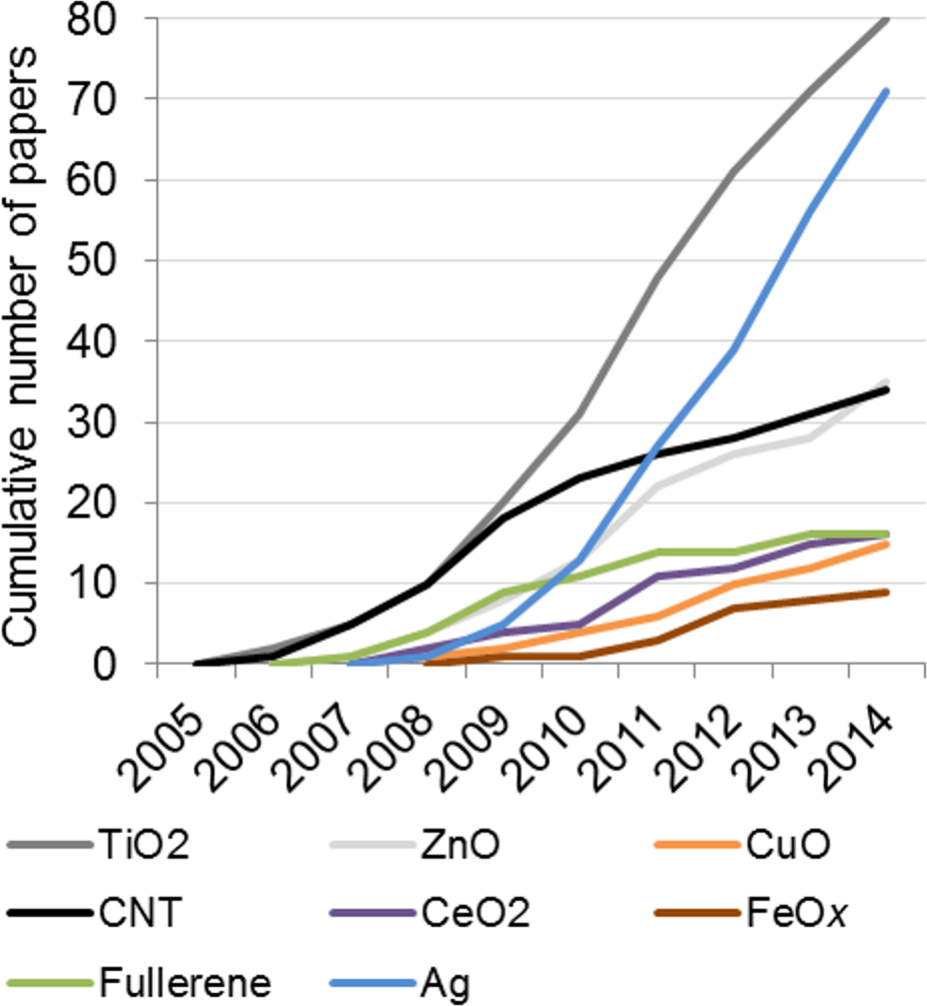
Evolution of nanoecotoxicological information about eight different nanomaterials according to the number of papers in NanoE-Tox database. The database entries were selected based on bibliometric data search in Thomson Reuters WoS using the keywords as indicated in Table 1 as of January 6, 2015.

Chronologically, the first nanoecotoxicological studies included in the database were published in 2006 and concerned TiO_2_ NPs and CNTs (Figure 3). The first papers on ecotoxicity of fullerenes and ZnO NPs were published in 2007 followed by CeO_2_, CuO and Ag NPs at 2008. While ecotoxicological effects of TiO_2_ are still extensively studied, the interest in ecotoxicology of CNTs has slightly decreased. Notably, the most rapid increase rate appears to be in the number of published papers about nanosilver (Figure 3). The information on ecotoxicity of FeO*_x_* particles started to emerge in 2009, i.e., later than for the other selected NPs (Figure 3). These findings are coherent with the literature survey by Kahru and Ivask [8] who showed that according to the citation pattern, the focus of the environment-related research shifted towards nanotoxicology by 2005 and the ‘pioneering’ NPs in environmental safety studies were CNTs, fullerenes, TiO_2_, SiO_2_ and ZnO. The analysis of the journals that contributed to the database revealed that more than half of the relevant papers originated from seven journals: Environmental Toxicology and Chemistry (29 papers), Environmental Science & Technology (25), Chemosphere (18), Environmental Pollution (12), Aquatic Toxicology (12), Science of the Total Environment (11), and Journal of Hazardous Materials (10 papers) (Table S4, Supporting Information File 2).

### Analysis of the database: physico-chemical characterisation of nanomaterials

**Figure 4:**
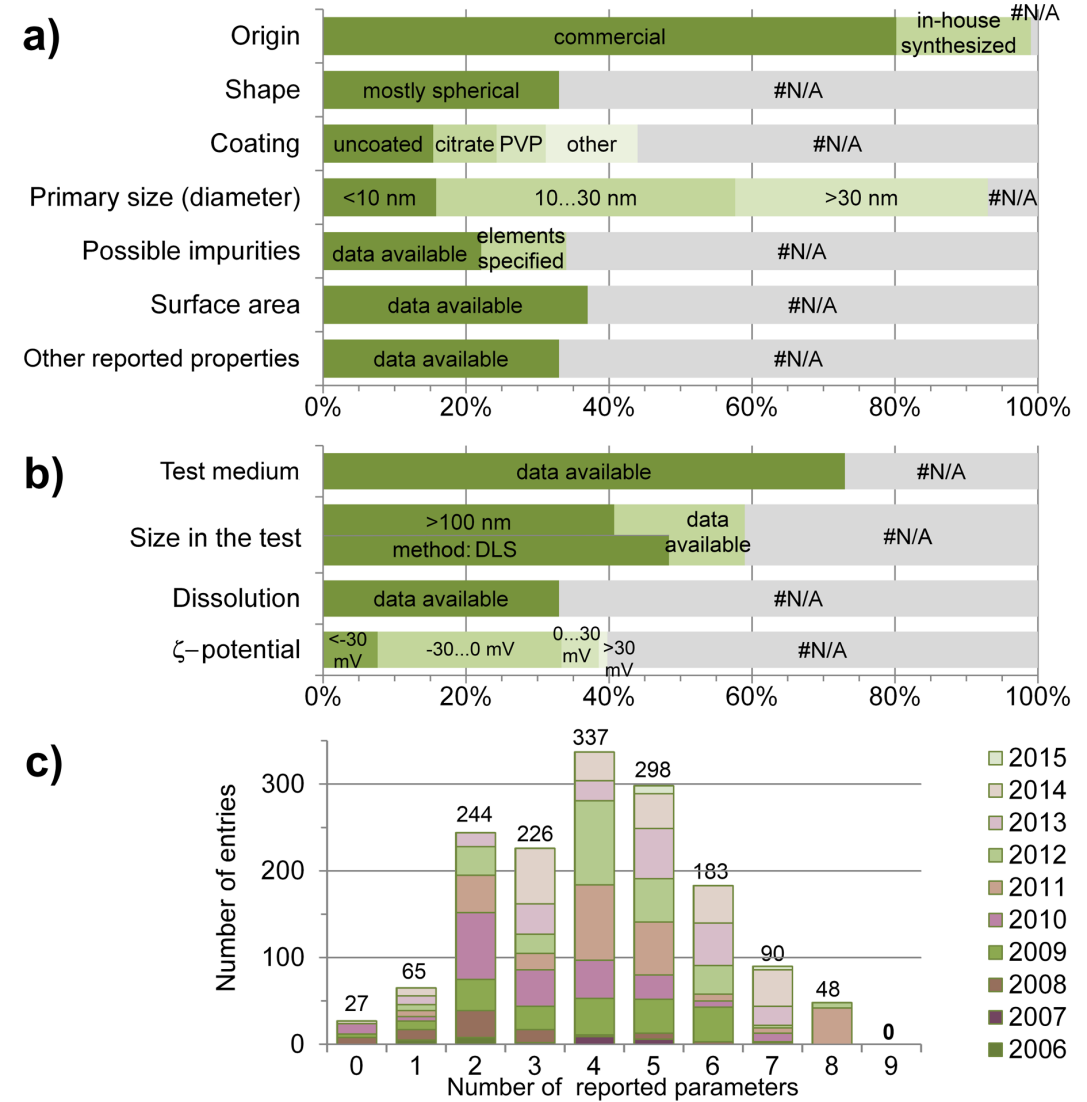
NanoE-Tox database: available data on characterisation of ENMs. Pristine (a) and environment-specific (b) properties as a percentage of all entries (1,518) in the database. Number of ENM parameters (shape, coating, primary size, impurities, surface area, other reported observations, size in the test, dissolution, ζ-potential) by number of entry and by publication year (c) #N/A - data not available. The database entries were selected based on bibliometric data search in Thomson Reuters WoS using the keywords as indicated in Table 1 as of January 6, 2015.

The physico-chemical characteristics of ENMs included in the NanoE-tox database can be divided to intrinsic properties and properties that are specific to the test environment. The intrinsic characteristics are: name, CAS number, origin, shape, initial coating or functionalization, primary size, possible impurities, surface area and other observations, and the test environment-specific characteristics are: media, size, dissolution and zeta potential (Supporting Information File 1). Figure 4 illustrates the distribution of the data on ENM characteristics in NanoE-Tox database. Analysis of the papers revealed that in 99% of the entries the origin of the ENMs was known and 80% of the nanomaterials were obtained from commercial sources (Figure 4a). The most common source for all ENMs was Sigma Aldrich, 40% of all commercial particles were obtained from there. TiO_2_ particles were mostly purchased from Evonik Industries (former Evonik-Degussa).

Many authors have emphasised that understanding the real risks of ENMs is a challenging task as there are several parameters that might have an influence on the biological effects of ENM [8,24,32–35]. Besides the chemical composition, the most important parameter determining the toxicity of NPs is their small size and size-dependent toxicity has been hypothesised in various papers [36–37]. Indeed, particle size has been considered as one of the most important physico-chemical parameter also in the papers collected in this study as this parameter was reported for 93% of the entries in the database. For all rod-shaped particles, also their length was reported. However, the results showed that most of the particles that were used in the 224 selected papers, were rather heterogeneous as in many cases the primary size was reported as a size range. According to Burello and Worth [38] ENMs with a diameter larger than 20–30 nm act often as bulk materials; thus, the “true nano-effects” are attributable to ENMs with smaller size. Indeed, in a recent paper on toxicity of different sizes of Ag NPs to bacteria, yeast, algae, crustaceans and mammalian cells in vitro Ivask et al. [39] showed that the toxicity of 20, 40, 60 and 80 nm monodisperse citrate-coated Ag NPs could fully be explained by released Ag ions whereas 10 nm Ag NPs proved more toxic than predicted. Analysis of the data in NanoE-Tox database revealed that the particles were smaller than 10 nm in 17% of the entries and in the size range of 10–30 nm in 45% of the entries (Figure 4a). Therefore, more than half of the studies have been performed using ENMs that should have size-dependent nanoeffects but as in most cases the NPs were polydisperse (i.e., had a broad size range) these effects were not often observed. Specific surface area that is closely related to the size of ENMs was reported in 37% of the entries (Figure 4a).

Another parameter that has been hypothesised to affect NP toxicity is morphology. For instance, some studies have shown that rod-shaped ENMs or triangular nanoplates could be more toxic than spherical ones [40–42]. However, the shape of ENMs was mentioned only in 33% of the entries and most of the experiments in the collected articles were performed with spherical particles (Figure 4a).

In addition to particle size and morphology, surface coating and/or functionalisation has been considered as an important parameter determining the biological effects of ENMs. For example, it has been discussed that coating on nanosilver plays an important role in Ag NPs toxicity [4,43–44]. However, information on initial coating or functionalisation of NPs was provided only in less than half of the entries. This is alarming because the surface chemistry of ENMs dictates their interactions with biological molecules and cells [45]. Altogether, 44% of the entries in the database contained information on NP coating: 29% of these were coated and 15% uncoated. ENMs were most often modified with citrate (31% of all coatings) and polyvinylpyrrolidone (PVP; 24% of all coatings) (Figure 4a). The high percentage of coated NPs in the database can be explained by the fact that nanosilver which constituted 35% of the database entries is frequently functionalised with different coatings, polyvinylpyrrolidone (PVP) and citrate being the most widely used.

A parameter closely related to NP surface properties is surface charge. It has been shown that positively charged ENMs tend to attach to the cellular surface that is negatively charged and these interactions may cause cell membrane damage [13,46]. In most studies ζ-potential is used as an indication of the surface charge of ENMs and NPs are considered to be stable in aqueous suspension if the ζ-potential is greater than ±30 mV [47]. In NanoE-Tox database, ζ-potential was reported in 40% of the entries. Most of the studies were performed with negatively charged ENMs (8% less than −30 mV, 25% −30…0 mV), 5% of the experiments were done with ENMs that had ζ-potential in the range of 0…+30 mV, and only 1% of the studies used stable positively charged ENMs (greater than +30 mV) (Figure 4b).

Another important parameter affecting toxicity of ENMs is the presence of impurities, for example presence of ‘seeding metals’ (catalysts) in CNTs that may count for observed toxic effects [48]. Purity of ENMs was reported in 34% of the entries; 65% of these cases mentioned purity as a percentage and 35% of the entries identified residual elements. Other reported observations, the most common parameters being crystal structure, density, and absorbance, were specified in 33% of the entries (Figure 4a).

Both in toxicological tests as well as in natural environments, the bioavailability and toxicity of ENMs depends on their fate in respective conditions [24,49]. In aquatic environment, ENMs tend to form agglomerates that might lead to their precipitation from the water phase; on the other hand, metal-based ENMs can release potentially toxic metal ions due to dissolution [50]. Cu^2+^, Zn^2+^ and Ag^+^, which can easily be released from respective ENMs are very toxic to a variety of aquatic organisms already at concentrations of milligrams and even micrograms per litre [4]. Analysis of the database entries (Figure 4b) showed that the most often reported ENM characteristic in the toxicity tests was hydrodynamic size (59% of all the entries) that usually (in 82% of the entries) was measured using dynamic light scattering (DLS) method. The data on hydrodynamic sizes indicated that ENMs tend to agglomerate in test conditions as 69% of the reported sizes were larger than 100 nm (in comparison, nearly all respective primary sizes were less than 100 nm). Dissolution of ENMs in toxicity tests was reported in 33% of all the entries. From all the studies using potentially soluble NPs (Ag, ZnO, CuO, CeO_2_ and FeO*_x_*) only half (51%) had measured the solubility of the particles.

As emphasised above, one of the goals of generating experimental nanotoxicological data is to apply them in model development that would allow for the comparison of physico-chemical properties of ENMs with their biological effects (QNAR models). It has been proposed that the QNAR models may even partially replace the expensive animal tests for evaluation of ENM related hazards [13]. Currently, there are a few QNAR modelling studies available for NPs [51]. However, these studies are based on relatively limited set of experimental data and therefore, applicable only for a small range of ENMs and organisms. Thus, in order to create a model with reasonable predictive power, several physico-chemical properties as well as data on a variety of NPs have to be included into the modelling to correlate the properties with toxic effects [25]. To evaluate whether the data in NanoE-Tox database might be suitable for (QNAR-)modelling, we analysed how many physico-chemical parameters of ENMs that could later be compared with the toxicological data were reported in each study. Nine physico-chemical parameters—shape, coating, primary size, impurities, surface area, other reported observations, size in the test, dissolution, surface charge (ζ-potential)—were analysed for the rate of being measured, i.e., how many of these were reported in one entry. In most of the studies, 2–6 of these parameters were reported (Figure 4c). Analysis of the data by year of publication revealed that despite of increasing number of nanotoxicological articles being published each year, some of these still report only up to three parameters of ENM. On the other hand, there were no studies where all nine selected physico-chemical properties were explored, and in only 9% of the studies 7–8 parameters were reported. Hence, although the ecotoxicological data on NPs are rapidly increasing, there is still a shortage of accompanying information concerning physico-chemical properties of ENMs that may limit the use of nano(eco)toxicological data for QNARs.

### Analysis of the database: ecotoxicological data

According to the European Union (EU) regulation on Registration, Evaluation, Authorisation and Restriction of Chemicals (REACH), the potential ecotoxicological effect of all chemical substances (including ENMs) that are produced in a volume of more than one tonne per year and sold in the EU must be evaluated. The amount of tests required depends on the production volume. If it exceeds 1 t/year, short-term tests with aquatic invertebrates (preferred species is *Daphnia*) and plants (algae is preferred) must be conducted. In case of the production volume over 10 t/year additional short-term tests with fish and studies of activated sludge respiration must be performed. Aforementioned aquatic studies must be performed also as long-term experiments for substances produced over 100 t/year; in addition, early life stage toxicity tests on fish, short-term toxicity tests on fish embryo and sac-fry stages and juvenile growth tests on fish must be carried out. With production over 100 t/year also terrestrial tests, short-term toxicity to invertebrates and plants and effects on soil microorganisms, must be performed. Finally, if the production volume for a certain substance exceeds 1,000 t/year, long-term terrestrial toxicity tests must be performed with invertebrates, plants, sediment organisms and birds in addition to all the previously mentioned aquatic and terrestrial studies [52].

To evaluate the compatibility of the toxicological data collected to NanoE-Tox database with the regulatory requirements, we collected the following data: type of test organism, test media, test duration and temperature, illumination conditions, test endpoint, toxicity measure and value. Also specific mechanisms of toxicity and accumulation of NPs in the cells, tissues or organs, and other observations were noted.

### Organisms used for evaluation of biological effects of ENMs

**Figure 5:**
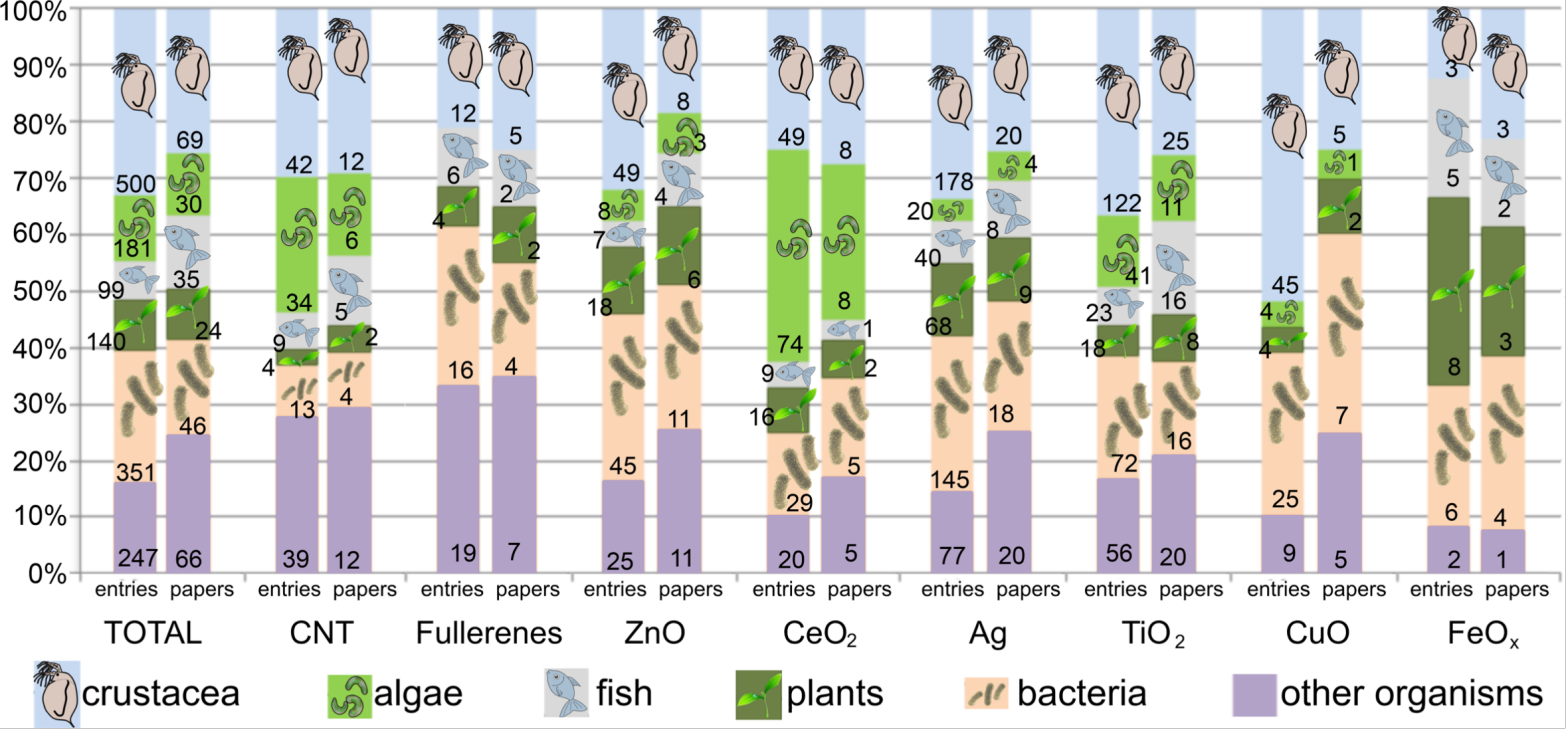
Types of test organisms used for evaluation of biological effects of selected ENMs in NanoE-Tox database. For each ENM, the left column represents the number of entries and right column represents the number of respective publications in the database. The database entries were selected based on bibliometric data search in Thomson Reuters WoS using the keywords as indicated in Table 1 as of January 6, 2015. The number of papers and entries for different ENMs is also presented in Figure 2.

Though the exact production volumes of ENMs are unknown, the estimated production of several ENMs exceeds the set 1 t/year limit [5]. Thus, according to legislation, several tests have to be conducted to bring these ENMs to the market. Organism-wise analysis of NanoE-Tox database revealed that information about effects of selected ENMs is available for 116 different test species (Table S5). Most of the experiments have been performed with water flea *Daphnia magna* (337 entries), followed by bacterium *Escherichia coli* (120 entries), unicellular alga *Pseudokirchneriella subcapitata* (107 entries), fish *Danio rerio* (66 entries), naturally luminescent bacterium *Vibrio fischeri* (44 entries), and nematode *Caenorhabditis elegans* (41 entries). In summary, by far the most often used test organisms were crustaceans constituting approximately one third (500/1,518) of all the tested species (Figure 5, Table S5, Supporting Information File 2). The abundance of toxicity data in crustaceans is likely derived from the mandatory reporting of these data according to REACH legislation as stated above. On the other hand, the amount of information about the effects of ENMs on algae – another mandatory test for REACH – is much more limited. With the keywords used in this study (Table 1), no information was found on algal toxicity of fullerenes and iron oxide and only one study evaluated the effect of CuO NPs on algae (Figure 5). The latter indicates that even if there are more publications on algal toxicity of ENMs, which were not retrieved in this study, the effects of ENMs on algae have been poorly studied. The same applies also to articles on effects of ENMs on fish. In NanoE-Tox database, there are no studies on the effect of CuO NPs on fish and only one study reported the effect of CeO_2_ NPs and two studies showed the effect of fullerenes and FeO*_x_* NPs to fish. Interestingly, toxicity tests with plants have been conducted with all 8 NPs. While relatively many studies have been performed with bacteria, the majority of them consider the effects towards potentially pathogenic bacterial strains, e.g., *Escherichia coli*, *Klebsiella pneumoniae*, *Pseudomonas aeruginosa*, *Staphylococcus aureus* (Table S5, Supporting Information File 2), which is likely driven by the important application area of some types of ENMs (TiO_2_, ZnO, CuO, Ag) as antimicrobials [4,53]. About 16% of the entries in the database regard test organisms other than crustaceans, algae, fish, plants and bacteria. Those organisms included yeasts, protists, amphibians, bivalves, cnidarians, echinoderms, insects, nematodes, rotifers, snails and worms (Table S5, Supporting Information File 2). Hence, quite a wide range of test organisms has already been included in the evaluation of biological effects of ENMs. This certainly increases environmental relevance of these studies and the NanoE-Tox database.

### Environmentally relevant test conditions

Recently, it has been highlighted that though most of the ENMs end up in the environment, relatively small amount of studies have been conducted in conditions relevant to the nature [54–56]. This was also reflected by the data collected into NanoE-Tox: 79% of the studies were performed in various artificial test media and only 15% in natural waters and 5% in soils, sludge or sediments. Generally, the test conditions were relatively well reported in the majority of the analysed papers: the time of exposure (test duration) was reported in nearly all cases, while the test temperature was documented in more than 90% of the entries and information about illumination (illumination conditions/dark) was mentioned in 75% of the entries.

#### Toxicity endpoints used

The toxicity values for ENMs, irrespective of the endpoint, were based on nominal concentrations of ENMs. As expected, in most of the studies (77% of the entries) the toxicological endpoint was viability (e.g., mortality, immobilisation, growth inhibition, luminescence/fluorescence inhibition) while the effects on viability were classically expressed as half-effective (EC_50_), half-inhibitory (IC_50_), or half-lethal (LC_50_) concentrations. 28% of the entries reported EC_50_ values, 10% LC_50_ values, 20% of the studies reported the concentration that did not exhibit any effect to the test organisms, i.e., NOEC (no observed effect concentration) values. However, some studies did not report any classical toxicity values because only one or two concentrations of NPs were tested by the authors; that did not allow for the establishment of a dose–response curve and, thus, calculations of E(L)C values. In addition, some papers considered the effect of ENMs on reproduction or studied possible malformations caused by ENMs that would be difficult to use for modelling purposes. As a result, the data that could be used as comparative inputs for models to evaluate the ecotoxicologial effects of ENMs is fairly limited in the database.

#### Analysis of the data consolidated into NanoE-Tox

Nano(eco)toxicological studies have usually two main aims: (i) the assessment of the toxic potential of ENMs, and (ii) the elucidation of the mechanism of toxic action [4,25]. In the following sections we will describe how NanoE-Tox database addresses these aims.

#### Toxicity of engineered nanomaterials

**Figure 6:**
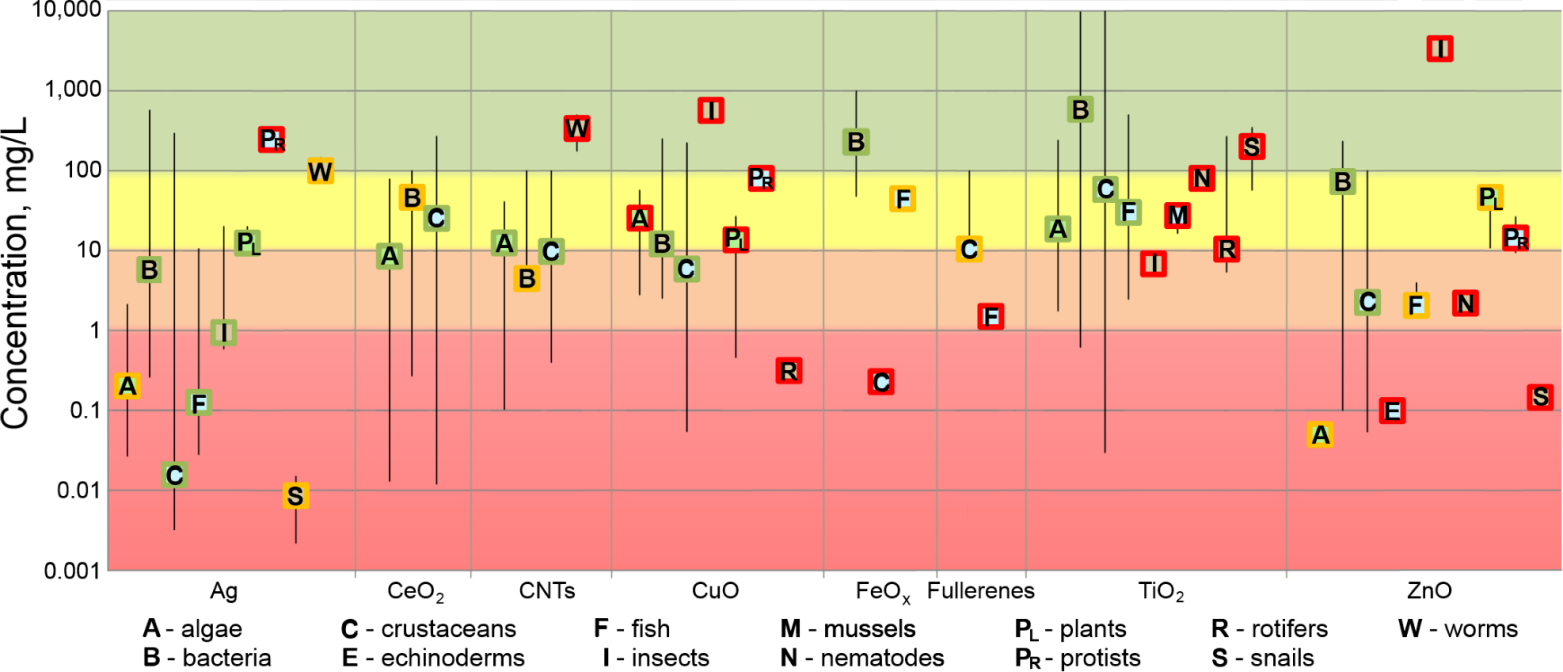
NanoE-Tox database: toxicity of selected nanoparticles to different organisms (data filtered by keyword ecotoxic*). Median E(L,I)C_50_ values ± minimum and maximum values. Colours of the frames surrounding the letters indicate the number of papers from which the respective data originates: red = 1 paper, orange = 2 papers, green ≥ 3 papers. The whiskers indicate the variability of the data. Note the logarithmic scale of y-axis. The E(L,I)C_50_ values used to derive the median values are from 113 papers and usually based on nominal concentration of the compound [44,55–56,61,64–172]. The toxicity ranking is indicated with the coloured background: E(L)C_50_ ≤ 1 mg/L – acutely very toxic, potentially chronically very toxic (red); E(L)C_50_ >1 to ≤10 mg/L – potentially chronically toxic (orange); E(L)C_50_ > 10 to ≤100 mg/L – potentially chronically harmful (yellow); E(L)C_50_ > 100 – not classified (green). The database entries were selected based on bibliometric data search in Thomson Reuters WoS^TM^ using the keywords as indicated in Table 1 as of January 6, 2015.

According to EU’s regulation on classification, labelling and packaging of substances and mixtures (CLP) [57], chemical substances can be categorised as acutely or chronically toxic based on the results of standardised toxicity tests (reviewed by Crane et al. [58]) with fish (96 h), crustaceans (48 h) or algae (72 or 96 h). While by legislation acute toxicity has only one category (E(L)C_50_ of the most sensitive organism ≤ 1 mg/L), chronic toxicity can be divided into four sub-categories (E(L)C_50_ ≤1 mg/L; E(L)C_50_ >1 to ≤10 mg/L; E(L)C_50_ > 10 to ≤100 mg/L; E(L)C_50_ > water solubility) that incorporate the degradation rate and bioconcentration factor of the chemical substance. Unfortunately, the latter two are not commonly determined in ecotoxicological studies; thus, in NanoE-Tox database bioconcentration factor has been reported only for FeO*_x_* in fish larvae [59] and TiO_2_ in coral tissue [60] and in crustaceans [61]. In order to give an overview of the ecotoxicity data collected for NanoE-Tox database (Figure 6), the hazard classification of ENMs was adjusted accordingly: acutely very toxic and potentially chronically very toxic (E(L)C_50_ ≤ 1 mg/L), potentially chronically toxic (E(L)C_50_ >1 to ≤10 mg/L), potentially chronically harmful (E(L)C_50_ > 10 to ≤100 mg/L) and not classified (E(L)C_50_ > 100). Figure 6 depicts median values of all EC_50_, LC_50_ and IC_50_ values with minimum and maximum values from NanoE-Tox database. Median EC_50_ values were calculated because these are the most precise estimates derived from the concentration–effect curve [62] and also, median EC_50_ values are often used in the QSAR analysis [63]. Analysis of the sources of the median values showed that most of the data in one data point originated from one (red frame, 19 points) or two (orange frame, 10 points) papers, only 18 median values were derived from 3 or more papers (green frame).

**Figure 7:**
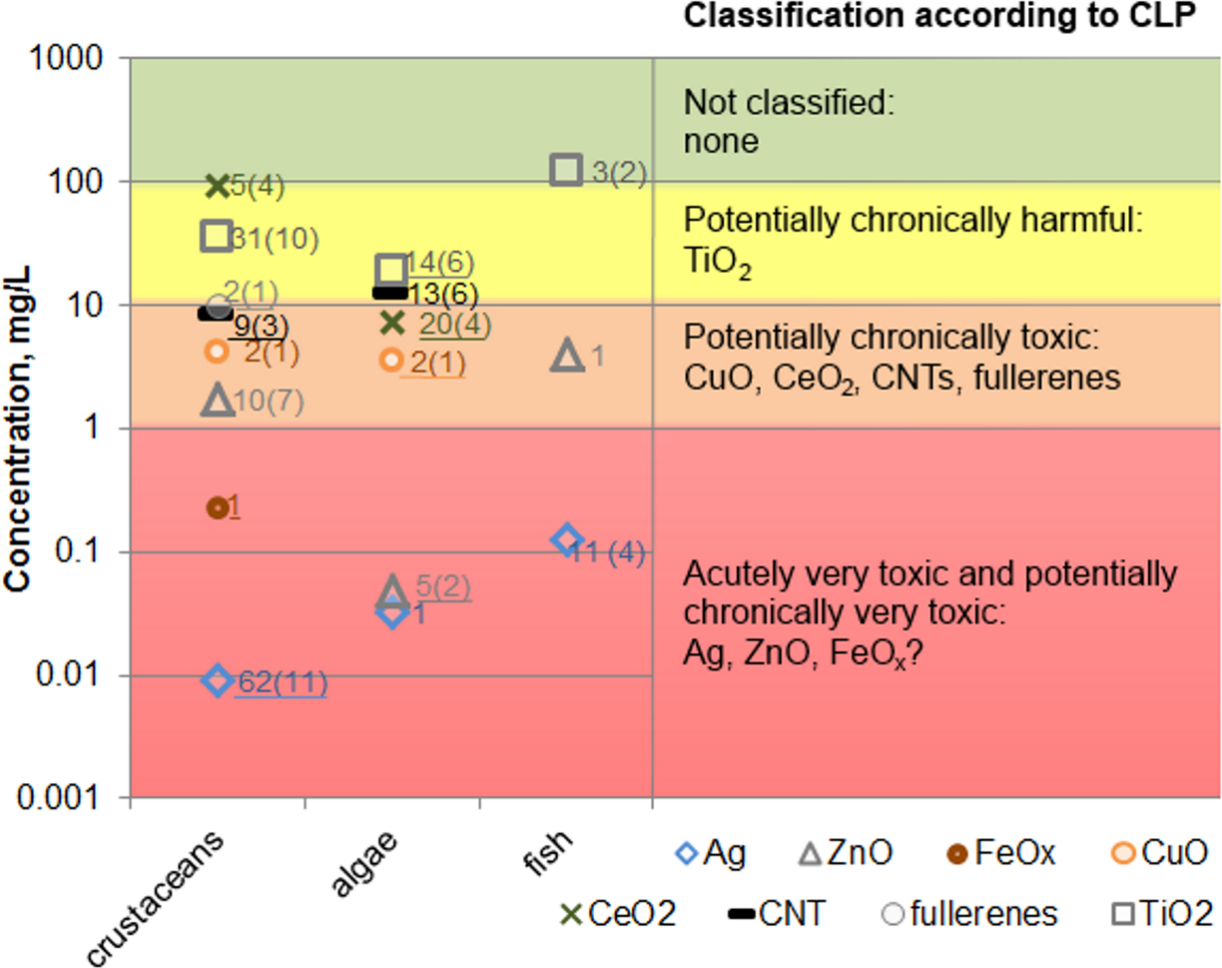
Classification of selected nanoparticles according to European Union CLP legislation based on their toxicity to fish (96 h), daphnids (48 h) and algae (72 or 96 h). Toxicity values were extracted from Figure 6. Classification of NPs is based on the most sensitive organism as described in CLP [57]. The number next to the symbol indicates the number of E(L,I)C_50_ values used to derive the median value and the number in the parenthesis indicates the number of papers from which the respective data originates. Underlined numbers indicate the datapoints (lowest E(L,I)C_50_ value for this ENM) used for classification. Note the logarithmic scale of the y-axis.

Based on the median toxicity values of the most sensitive organisms (i.e., theoretically representing the weakest link in the ecosystem), the toxicity of selected ENMs decreased in the order Ag > ZnO > FeO*_x_* > CuO > fullerenes > CNTs > TiO_2_ > CeO_2_. However, when toxicity values that were derived from three or more papers were considered, the order slightly changed: Ag > ZnO > CuO > CeO_2_ > CNTs > TiO_2_ > FeO*_x_*. The median values reported here are in general agreement with those published previously [4,24,26] (Table 2). However, such evaluation where the median values are derived across all different test conditions and test species is not in accordance with the current legislation. In order to be coherent with legislation, we next analysed the toxicity data obtained in standard tests with fish (96 h), daphnids (48 h) and algae (72 or 96 h) (Figure 7), i.e., the mandatory tests required under CLP [57] for classification of substances, and applied the same hazard ranking criteria as was used in Figure 6. This analysis showed that the most toxic ENM was Ag that could be classified as “acutely very toxic” and “potentially chronically very toxic”. ZnO and FeO*_x_* were also ranked as “acutely very toxic” and “potentially chronically very toxic” although less toxic than Ag. It is worth mentioning that the classification of FeO*_x_* NPs was based on only one study (entry in the database), warranting further research of FeO*_x_* NPs for more accurate ecotoxicity evaluation. According to median E(L)C_50_ values from the standard toxicity tests, CuO and CeO_2_ NPs, CNTs and fullerenes fell into the category of “potentially chronically toxic” and TiO_2_ NPs were ranked as “potentially chronically harmful”.

**Table 2:** Comparison of the median E(L,I)C_50_ values for different species in NanoE-Tox database and previous reviews [4,24,26].

ENM	E(L,I)C_50_ range in NanoE-Tox	E(L,I)C_50_ range in other reviews

Ag	0.01–245 mg/L	0.01–38 mg/L [4] 0.04–39 mg/L [24]
CeO_2_	8.5–46.6 mg/L	0.1–100 mg/L [26]
CNTs	4.5–338 mg/L	1.0–500 mg/L [24]
CuO	0.32–569 mg/L	2.1–100 mg/L [4] 0.71–127 mg/L [24]
FeO*_x_*	0.23–240 mg/L	#N/A^a^
fullerenes	1.5–11 mg/L	0.25–100 mg/L [24]
TiO_2_	6.8–589 mg/L	39–11987 mg/L [24]
ZnO	0.05–3376 mg/L	0.08–121 mg/L [4] 0.055–97.4 mg/L [24]

^a^ #N/A: not applicable.

#### Mechanism of toxic action

While after a decade-long research the exact mechanisms of toxic action of ENMs are still debated, the main proposed mechanisms can be outlined as follows: (i) physical interactions of ENMs with cells or cellular components, (ii) production of reactive oxygen species and resulting induction of oxidative stress, and (iii) toxic effect of released ions from metal/metal oxide ENMs [13,25,28]. Analyses of the information in NanoE-Tox database (Table S2, Supporting Information File 2) revealed that the most often reported potential mechanism of toxic action for ZnO [128–132,173], Ag [44,64–73,174–177], and CuO [55,64,73,126–129,173] NPs was the release of metal ions. On the other hand, some studies have also proposed that the toxicity of these ENMs might be at least partially caused by the NPs themselves [73–84,178–181]. However, most of the studies reporting NP-specific effects of Ag, CuO and ZnO used insoluble particles and tested them in higher concentrations compared to the ones commonly reported as toxic. Thus, it can be concluded, in accordance with some previous studies [4,25], that in most cases the observed toxicity of these three ENMs was triggered by toxic metal ions. Other modes of toxic action reported for Ag NPs included destabilisation of cell membranes/mechanical membrane damage [89,175,182–183], oxidative stress [71,73,89,175–176,184–185], DNA damage/genotoxicity [102,186–187], and binding to sulfhydryl groups [100]. Similar effects were also demonstrated in case of ZnO NPs [84–86,188–190]. The mechanism of toxic action of insoluble ENMs like CeO_2_ [109–110], CNTs [116,133,191] and TiO_2_ [153–156,192] was usually reported as particle-driven mechanical membrane damage. NanoE-Tox database contains only one study suggesting the mechanism of toxicity of fullerenes (oxidative stress) [193] and there are no data about possible mechanism of action of FeO*_x_* NPs.

Additionally, the information collected to the NanoE-Tox database indicated that ENMs were readily ingested by different organisms [55,72,77,119–123,192,194–202] and tended to accumulate in them [55,59–60,69–71,84,122–126,159,176–179,187,189,192,201–214] or on their surface [79,117–119,126,136–140,196,215–218] (Table S3, Supporting Information File 2). Similar findings have been reported in previous studies [24–26,29].

## Conclusion

NanoE-Tox database that is available as Supporting Information File 1 of this paper is the first online-available database that contains in-depth nanoecotoxicological information on eight ENMs accompanied by considerable amount of information on ENM physico-chemical properties, testing conditions and, to some extent, also on mechanisms of toxic action. Hence, NanoE-Tox enables the comparison of toxicity of ENMs across different test species and, in addition, could provide valuable input for computational toxicity modeling (e.g., QSARs) and risk assessment.

The analysis of the database entries resulted in coherent data with previously published studies: the most toxic of the selected ENMs were Ag NPs followed by ZnO and CuO NPs and the toxicity of these ENMs was largely triggered by their solubility. Additionally, systematic collection of the data revealed several gaps in the current knowledge about ENM ecotoxicity: (i) in most cases the physico-chemical properties of the investigated NPs were described insufficiently, (ii) relatively few experiments have been performed with algae and fish, and (iii) ecotoxicity tests with standard test organisms were often performed with modified protocols (i.e., duration of the test was either shorter or longer than required by the OECD or ISO standards). Although the NanoE-Tox database is limited to a selected range of articles entered in the Thomson Reuters WoS database by January 6, 2015 and retrieved by using specific keywords, it provides a good overview of the existing ecotoxicological information about Ag, CeO_2_, CuO, FeO*_x_*, TiO_2_ and ZnO NPs, carbon nanotubes and fullerenes.

## Supporting Information

File 1 Supplementary tables.

File 2 NanoE-Tox database.
