# Supplementary material for: NanoE-Tox: New and in-depth database concerning ecotoxicity of nanomaterials
Source: Beilstein J Nanotechnol. 2015 Aug 25;6:1788–804. doi: 10.3762/bjnano.6.183 (PMC4578397; doi:10.3762/bjnano.6.183)
Supplement: File 1 — Supplementary tables. [file Beilstein_J_Nanotechnol-06-1788-s001.pdf]

Supporting Information

for

NanoE-Tox: New and in-depth database concerning ecotoxicity of nanomaterials

Katre Juganson[1,2*], Angela Ivask[1,3], Irina Blinova[1], Monika Mortimer[1,4], and Anne Kahru[1]

Address: [1]Laboratory of Environmental Toxicology, National Institute of Chemical Physics and Biophysics, Akadeemia tee 23, 12618 Tallinn, Estonia; [2]Department of Chemistry, Tallinn University of Technology, Akadeemia tee 15, 12618 Tallinn, Estonia; [3]Mawson Institute, University of South Australia, Mawson Lakes, 5095 South Australia, Australia and [4]Bren School of Environmental Science & Management, University of California Santa Barbara, Santa Barbara, California 93106-5131, United States

Email: Katre Juganson* - katre.juganson@kbfi.ee

* Corresponding autor:

Supplementary tables

Table S1: Total number of publications in Thomson Reuters Web of Science™ for different applications of eight selected nanomaterials. Search was performed combining the keywords listed in the table with light grey background. The search was done on March 19th, 2015.

nano* AND	Ag NPs	CeO_2 NPs	CNTs	CuO NPs	FeO_x NPs	Fullerenes	TiO_2 NPs	ZnO NPs	Total
**Fields of application**	silver OR Ag	cerium *oxide OR ceria OR CeO2	carbon nanotu* OR CNT OR *CNT	copper oxide OR CuO	iron *oxide OR Fe3O4 OR Fe2O3	fulleren*	titanium *oxide OR titania OR TiO2	zinc oxide OR ZnO	
Hydrogen storage	189	124	3642	64	195	357	314	110	**1040**
Environmental remediation	110	16	155	36	219	23	303	74	**655**
Catalysis	2043	624	2553	458	1225	169	2652	711	**5215**
Drug delivery	999	55	2966	60	3687	272	515	284	**4818**
Medical imaging	97	7	151	4	292	19	32	21	**368**
Photovoltaics	299	3	368	41	14	355	632	401	**1443**
Textiles	568	11	482	44	77	14	650	305	**1090**
Therapeutics	109	16	324	8	322	35	32	17	**414**
Reinforced composites	172	37	4161	53	83	91	260	102	**589**
Electronics	1080	16	3331	113	116	286	278	542	**1335**
Optics	987	20	638	35	120	89	249	256	**749**
Coatings and pigments	19	12	10	8	36	1	143	53	**241**
Cosmetics	133	24	95	8	36	28	265	148	**485**
Ceramics applications	186	140	413	53	132	17	578	202	**982**
Anti-oxidants	10	13	14	4	4	7	3	5	**23**
Lubrication	78	10	181	26	37	115	55	35	**268**
Sensors	5327	288	13004	977	1421	255	2596	4159	**9408**
Absorbents	37	9	158	10	77	4	45	35	**171**
Energetics	136	18	801	11	38	267	218	54	**588**
Magnetics	84	3	154	29	1156	13	51	53	**1302**
Water purification	304	33	905	56	468	60	782	142	**1508**
Air emissions reduction	18	9	23	7	9	3	19	23	**61**
Natural and green products	36	1	11	2	10	0	6	6	**24**
Quantum computing	31	5	282	1	52	49	26	20	**148**
Masonry and building materials	1	0	0	0	0	0	1	0	**1**
Photonics	297	2	322	17	31	31	87	152	**318**
Surfactants	689	97	1166	135	468	80	506	335	**1524**
Antimicrobials	2931	13	299	120	154	52	504	341	**1171**
**Total**	**16970**	**1606**	**36609**	**2380**	**10479**	**2692**	**11802**	**8586**	**35939**

Table S2: Mechanisms of toxic action of selected ENMs in different organisms based on information in NanoE-Tox database.

	Ag	CeO_2	CNTs	CuO	Fullerenes	TiO_2	ZnO
Released ions	bacteria [1-5]<br>bivalves [6]<br>crustaceans [7-9]<br>earthworms [10]<br>plants [11-13]<br>protists [14]<br>yeasts [15]			bacteria [5,16-19]<br>crustaceans [8,20]<br>insects [21]			bacteria [16-18,22]<br>crustaceans [23]<br>echinoderms [24]
Effect of NPs/ their primary size	bacteria [25]<br>crustaceans [26,27]			algae [28]<br>plants [29]		algae [30]<br>crustaceans [30,31]<br>nematodes [32]<br>rotifers [30]	plants [33]
Ions + NPs	algae [34]<br>crustaceans [35]<br>fish [36,37]<br>nematodes [38]			protists [39]<br>snails [40]			bacteria [5]<br>crustaceans [41]<br>fish [42]
Destabilization of cell membranes/ mechanical membrane damage	bacteria [43,44]<br>earthworms [45]<br>yeasts [15]	algae [46,47]	algae [48]<br>bacteria [49]<br>crustaceans [50]	protists [39]		bacteria [51]<br>fish [52]	algae [53]<br>fish [42]
Oxidative stress	bacteria [5,43,54]<br>insects [55]<br>bivalves [6]<br>plants [12]<br>yeasts [15]	nematodes [56]	algae [48,57]<br>crustaceans [50]		crustaceans [58]	bacteria [43,51]<br>fish [52,59]<br>nematodes [32]	bacteria [60]<br>earthworms [61]<br>fish [62]<br>snails [63]
DNA damage/ genotoxic	crustaceans [64]<br>insects [65]<br>plants [66]						bacteria [60]<br>earthworms [61]
Disturbing ATP production			algae [57]				
Shading effect		algae [46]	algae [48,67]				
Effect of accumulation on the organisms	fish [36]	algae [46]<br>crustaceans [68,69]	algae [67]			crustaceans [70]	fish [42]
Binding to -SH groups	fish [71]						

*There was no information about mechanisms of toxic action of FeO_x in the NanoE-Tox database

Table S3: Accumulation and uptake of selected ENMs in different organisms based on information in NanoE-Tox database.

	Ag	CeO_2	CNTs	CuO	FeO_x	Fullerenes	TiO_2	ZnO
Accumulated in organisms	bacterial biofilm [72]<br>bivalves [6]<br>crustaceans [73]<br>earthworms [10]<br>plants [11-13,66,74]		amphibians [75]<br>bivalves [76]<br>crustaceans [77,78]	bivalves [79]<br>crustaceans [20]<br>insects [21]<br>snails [40]<br>plant roots [29]	crustaceans [80]<br>fish [81]	blackworms [82]<br>crustaceans [83,84]	corals [85]<br>crustaceans [86]<br>fish [87-90]<br>nematodes [32]<br>plants [91,92]	earthworms [61]<br>fish [42]
Accumulated on surface of organisms	crustaceans [93]<br>fish eggs [37]	crustaceans [69]<br>plant roots [94]	algae [67]<br>bivalves [95]<br>crustaceans [95-97]<br>plant roots [98]	insects [21]			bacteria [99]<br>crustaceans [70]<br>plants [100]	algae [101]<br>plant roots [102]
Ingested by organisms	crustaceans [26]<br>protists [14]	nematodes [56]	amphibians [103]<br>bivalves [76,95]<br>crustaceans [77,84,95,96,104,105]<br>protists [106]	crustaceans [20]	crustaceans [80]	crustaceans [84]	crustaceans [84,107,108]<br>lugworms [109]<br>nematodes [32]	crustaceans [84]
Translocation seed → plant (yes/no)			no [98]					
Translocation root → shoot (yes/no)		no/very low [94]		limited [29]		yes [98]	yes if size is less than 36 nm [92]	very low [102]
Other observations					BCF in fish larvae 0.04…0.14 [81]		Significant uptake in *E. coli; [60]*<br>Dose-dependent increase in internalization in bacteria; [110]<br>BCF in coral tissue 2…62, in posterior mixture 238…594; [85]<br>BCF in crustaceans under illumination 502, in dark 318 [111]	Significant uptake in *E. coli; [60]*<br>Dose-dependent increase in internalization in bacteria [110]

Table S4: List of journals in NanoE-Tox.

	Journal title	No. of papers	No. of entries	5-year impact factor
1	ENVIRONMENTAL TOXICOLOGY AND CHEMISTRY	29	156	3.282
2	ENVIRONMENTAL SCIENCE & TECHNOLOGY	25	170	6.277
3	CHEMOSPHERE	18	129	3.897
4	ENVIRONMENTAL POLLUTION	12	107	4.306
5	AQUATIC TOXICOLOGY	12	31	3.948
6	SCIENCE OF THE TOTAL ENVIRONMENT	11	85	3.906
7	JOURNAL OF HAZARDOUS MATERIALS	10	79	5.123
8	PloS one	7	34	4.015
9	ECOTOXICOLOGY AND ENVIRONMENTAL SAFETY	6	73	2.715
10	NANOTOXICOLOGY	5	23	7.766
11	ENVIRONMENTAL SCIENCE AND POLLUTION RESEARCH	5	62	2.951
12	ECOTOXICOLOGY	4	16	3.191
13	JOURNAL OF NANOPARTICLE RESEARCH	4	31	2.927
14	ENVIRONMENTAL TOXICOLOGY	4	50	2.371
15	JOURNAL OF ENVIRONMENTAL MONITORING	4	10	2.133
16	ENVIRONMENTAL TOXICOLOGY AND PHARMACOLOGY	4	28	2.093
17	ACS Nano	3	26	13.774
18	NANOMEDICINE	3	26	5.966
19	TOXICOLOGICAL SCIENCES	3	38	4.855
20	JOURNAL OF NANOBIOTECHNOLOGY	3	19	#N/A
21	ANALYTICAL AND BIOANALYTICAL CHEMISTRY	2	65	3.744
22	DESALINATION	2	29	3.481
23	MARINE ENVIRONMENTAL RESEARCH	2	10	2.525
24	JOURNAL OF ENVIRONMENTAL SCIENCES-CHINA	2	3	2.465
25	ARCHIVES OF ENVIRONMENTAL CONTAMINATION AND TOXICOLOGY	2	6	2.135
26	TOXICOLOGICAL AND ENVIRONMENTAL CHEMISTRY	2	24	#N/A
27	NATURE NANOTECHNOLOGY	1	5	38.586
28	SMALL	1	4	8.416
29	CARBON	1	7	6.638
30	WATER RESEARCH	1	6	6.092
31	FREE RADICAL BIOLOGY AND MEDICINE	1	2	5.983
32	SOIL BIOLOGY & BIOCHEMISTRY	1	1	4.785
33	LANGMUIR	1	6	4.489
34	COLLOIDS AND SURFACES B-BIOINTERFACES	1	14	4.226
35	APPLIED MICROBIOLOGY AND BIOTECHNOLOGY	1	12	4.138
36	ENVIRONMENTAL RESEARCH	1	4	4.033
37	TOXICOLOGY	1	9	3.884
38	TOXICOLOGY LETTERS	1	4	3.706
39	ENVIRONMENTAL MICROBIOLOGY REPORTS	1	3	3.556
40	ECOLOGICAL ENGINEERING	1	2	3.479
41	CYTOMETRY PART A	1	2	3.306
42	FOOD AND CHEMICAL TOXICOLOGY	1	1	3.21
43	PROCESS BIOCHEMISTRY	1	2	2.922
44	MUTATION RESEARCH-GENETIC TOXICOLOGY AND ENVIRONMENTAL MUTAGENESIS	1	4	2.716
45	COLLOIDS AND SURFACES A-PHYSICOCHEMICAL AND ENGINEERING ASPECTS	1	10	2.494
46	HYDROBIOLOGIA	1	3	2.35

	Journal title	No. of papers	No. of entries	5-year impact factor
47	PARASITOLOGY RESEARCH	1	2	2.286
48	APPLIED BIOCHEMISTRY AND BIOTECHNOLOGY	1	1	1.994
49	WATER AIR AND SOIL POLLUTION	1	1	1.943
50	ANALYTICAL METHODS	1	1	1.913
51	JOURNAL OF TOXICOLOGY AND ENVIRONMENTAL HEALTH-PART A-CURRENT ISSUES	1	2	1.868
52	BIOLOGICAL TRACE ELEMENT RESEARCH	1	1	1.656
53	JOURNAL OF MICROBIOLOGY AND BIOTECHNOLOGY	1	2	1.484
54	SCIENTIFIC WORLD JOURNAL	1	12	1.3
55	JOURNAL OF ENVIRONMENTAL SCIENCE AND HEALTH PART A-TOXIC/HAZARDOUS SUBSTANCES & ENVIRONMENTAL ENGINEERING	1	4	1.233
56	BULLETIN OF THE KOREAN CHEMICAL SOCIETY	1	2	0.797
57	ASIAN PACIFIC JOURNAL OF TROPICAL MEDICINE	1	4	0.665
58	ARCHIVES OF BIOLOGICAL SCIENCES	1	4	0.606
59	INLAND WATER BIOLOGY	1	17	0.321
60	ENVIRONMENTAL ENGINEERING RESEARCH	1	3	#N/A
61	JOURNAL OF ENVIRONMENTAL HEALTH SCIENCES	1	16	#N/A
62	ACS SUSTAINABLE CHEMISTRY & ENGINEERING	1	3	#N/A
63	ENVIRONMENTAL HEALTH AND TOXICOLOGY	1	3	#N/A
64	ENVIRONMENTAL SCIENCE-PROCESSES & IMPACTS	1	2	#N/A
65	NANOCON 2009, CONFERENCE PROCEEDINGS	1	6	#N/A
66	NANOSAFE 2012: INTERNATIONAL CONFERENCES ON SAFE PRODUCTION AND USE OF NANOMATERIALS	1	1	#N/A
	**TOTAL**	224	1518	

Table S5: Organism-wise distribution of data in NanoE-Tox. Number in parenthesis indicates number of entries in the database.

		CNT	Fullerenes	ZnO	CeO2	Ag	TiO2	CuO	FeOx
Algae	Green algae	*Chlorella sp.* (3)<br>*Chlorella vulgaris* (18)<br>*Dunaliella tertiolecta* (2)<br>*Pseudokirchneriella subcapitata* (7)		*Pseudokirchneriella subcapitata* (5)	*Chlamydomonas reinhardtii* (4)<br>*Pseudokirchneriella subcapitata* (68)	*Chlorella vulgaris* (2)<br>*Dunaliella tertiolecta* (2)<br>*Pseudokirchneriella subcapitata* (4)	*Chlamydomonas moewusii* (1)<br>*Chlamydomonas reinhardtii* (1)<br>*Chlorella sp* (2)<br>*Chlorella vulgaris* (2)<br>*Desmodesmus subspicatus* (4)<br>*Phaeodactylum tricornutum* (3)<br>*Pseudokirchneriella subcapitata* (23)<br>*Scenedesmus sp* (2)<br>*Scenedesmus obliquus* (2)	*Chlorella spp* (2)<br>*Nitellopsis obtusa* (2)	
	Red algae	*Thalassiosira pseudonana* (4)		*Thalassiosira weissflogii* (3)	*Nitzschia palea* (2)	*Ceramium tenuicorne* (12)	*Scenedesmus quadricauda* (1)		
Amphibians		*Ambystoma mexicanum* (1)<br>*Xenopus laevis* (13)			*Pleurodeles waltl* (2)<br>*Xenopus laevis* (3)				
Bacteria		*Bacillus subtilis* (2)<br>*Cupriavidus metallidurans* (1)<br>*Escherichia coli* (4)<br>*Pseudomonas aeruginosa* (2)<br>*Staphylococcus aureus* (2)<br>*Vibrio fischeri* (1)	*Bacillus stearothermophilus* (1)<br>*Escherichia coli* (12)<br>*Vibrio fischeri* (2)	*Anabaena flos-aquae* (3)<br>*Escherichia coli* (24)<br>*Pseudomonas putida* (7)<br>*Vibrio fischeri* (6)	*Anabaena* (24)<br>*Escherichia coli* (1)<br>*Vibrio fischeri* (3)	*Arthrobacter globiformis* (2)<br>*Bacillus aquimaris* (12)<br>*Bacillus thuringiensis* (12)<br>*Enterococcus faecalis* (1)<br>*Escherichia coli* (27)<br>*Klebsiella pneumoniae* (1)<br>*Nitrosomonas europaea* (2)<br>*Pseudomonas aeruginosa* (2)<br>*Pseudomonas chlororaphis* (10)<br>*Pseudomonas putida* (16)<br>*Staphylococcus aureus* (2)<br>*Staphylococcus epidermidis* (1)<br>*Vibrio fischeri* (4)	*Anabaena variabilis* (1)<br>*Cupriavidus metallidurans* (6)<br>*Escherichia coli* (37)<br>*Pseudomonas putida* (2)<br>*Vibrio fischeri* (18)	*Escherichia coli* (13)<br>*Vibrio fischeri* (7)	*Escherichia coli* (2)<br>*Vibrio fischeri* (3)
Bivalves	Clams					*Macoma balthica* (2)		*Macoma balthica* (1)	
	Mussels	*Mytilus galloprovincialis* (2)<br>*Villosa iris* (2)	*Mytilus galloprovincialis* (2)			*Elliptio complanata* (2)	*Dreissena polymorpha* (1)<br>*Mytilus galloprovincialis* (4)		
Cnidarians	Corals					*Acropora japonica* (8)	*Montastraea faveolata* (2)		
	Hydra	*Hydra attenuata* (1)	*Hydra attenuata* (1)				*Hydra attenuata* (1)		
Crustaceans	Amphipods	*Hyalella azteca* (4)<br>*Leptocheirus plumulosus* (1)					*Gammarus fossarum* (7)		
	Branchiopods	*Ceriodaphnia dubia* (11)<br>*Daphnia magna* (15)<br>*Daphnia similis* (2)<br>*Thamnocephalus platyurus* (1)	*Daphnia magna* (7)<br>*Daphnia pulex* (4)<br>*Thamnocephalus platyurus* (1)	*Ceriodaphnia affinis* (4)<br>*Daphnia magna* (28)<br>*Thamnocephalus platyurus* (10)	*Ceriodaphnia affinis* (4)<br>*Ceriodaphnia dubia* (1)<br>*Chydorus sphaericus* (1)<br>*Daphnia magna* (34)<br>*Daphnia pulex* (4)<br>*Daphnia similis* (2)<br>*Thamnocephalus platyurus* (3)	*Artemia nauplii* (12)<br>*Chydorus sphaericus* (3)<br>*Daphnia galeata* (3)<br>*Daphnia magna* (141)<br>*Daphnia pulex* (3)<br>*Thamnocephalus platyurus* (8)	*Artemia salina* (8)<br>*Ceriodaphnia affinis* (4)<br>*Ceriodaphnia dubia* (3)<br>*Chydorus sphaericus* (1)<br>*Daphnia magna* (78)<br>*Daphnia similis* (17)<br>*Thamnocephalus platyurus* (4)	*Daphnia magna* (33)<br>*Thamnocephalus platyurus* (12)	*Ceriodaphnia dubia* (2)<br>*Daphnia magna* (1)
	Copepods	*Amphiascus tenuiremis* (2)<br>*Tigriopus japonicus* (6)		*Acartia tonsa* (6)		*Tisbe battagliai* (8)			
	Ostracods			*Heterocypris incongruens* (1)					
Fish		*Danio rerio* (2)<br>*Oncorhynchus mykiss* (3)<br>*Oreochromis niloticus* (1)<br>*Oryzias melastigma* (2)	*Danio rerio* (5)	*Cyprinus carpio* (1)<br>*Danio rerio* (6)	*Danio rerio* (9)	*Danio rerio* (30)<br>*Oncorhynchus mykiss* (4)<br>*Oryzias latipes* (2)<br>*Pimephales promelas* (4)	*Cyprinus carpio* (4)<br>*Danio rerio* (10)<br>*Oncorhynchus mykiss* (3)<br>*Oryzias latipes* (6)		*Danio rerio* (4)<br>*Oryzias latipes* (1)
Insects		*Chironomus dilutus* (3)<br>*Drosophila melanogaster* (2)	*Drosophila melanogaster* (1)	*Folsomia candida* (6)	*Chironomus riparius* (6)	*Aedes aegypti* (2)<br>*Chironomus riparius* (7)<br>*Culex quinquefasciatus* (2)<br>*Drosophila melanogaster* (4)<br>*Folsomia candida* (2)	*Bovicola ovis* (1)<br>*Chironomus riparius* (2)<br>*Hippobosca maculata* (1)	*Allogamus ligonifer* (2)	
Nematodes				*Caenorhabditis elegans* (1)	*Caenorhabditis elegans* (7)	*Caenorhabditis elegans* (18)	*Caenorhabditis elegans* (15)		
Plants		*Cucurbita pepo* (1)<br>*Oryza sativa* (3)	*Lemna gibba* (3)<br>*Oryza sativa* (1)	*Cucurbita pepo* (2)<br>*Fagopyrum esculentum* (2)<br>*Lactuca sativa* (1)<br>*Lepidium sativum* (10)<br>*Lolium perenne* (1)<br>*Raphanus sativus* (1)<br>*Vicia faba* (1)	*Cucumis sativus* (3)<br>*Cucurbita maxima* (3)<br>*Lactuca sativa* (3)<br>*Solanum lycopersicum* (3)<br>*Spinacia oleracea* (1)<br>*Triticum aestivum* (3)	*Allium cepa* (2)<br>*Arabidopsis thaliana* (3)<br>*Cucumis sativus* (1)<br>*Cucurbita pepo* (9)<br>*Hordeum vulgare* (10)<br>*Lactuca sativa* (2)<br>*Lemna gibba* (4)<br>*Linum usitatissimum* (6)<br>*Lolium perenne* (7)<br>*Lycopersicum esculentum* (10)<br>*Nicotiana tabacum* (2)<br>*Zea mays* (12)	*Cucumis sativus* (1)<br>*Lactuca sativa* (3)<br>*Lemna minor* (3)<br>*Lepidium sativum* (3)<br>*Raphanus sativus* (1)<br>*Solanum lycopersicum* (1)<br>*Spinacia oleracea* (1)<br>*Triticum aestivum* (5)	*Lactuca sativa* (1)<br>*Raphanus sativus* (1)<br>*Schoenoplectus tabernaemontani* (2)	*Cucumis sativus* (2)<br>*Lactuca sativa* (3)<br>*Raphanus sativus* (1)<br>*Solanum lycopersicum* (1)<br>*Spinacia oleracea* (1)
Protists		*Tetrahymena thermophila* (5)		*Bodo saltans* (1)<br>*Euglena gracilis* (3)<br>*Tetrahymena thermophila* (4)	*Bodo saltans* (2)	*Tetrahymena thermophila* (2)	*Bodo saltans* (2)	*Tetrahymena thermophila* (2)	
Rotifers							*Brachionus plicatilis* (3)	*Brachionus calyciflorus* (2)	
Echinoderms				*Lytechinus pictus* (1)			*Lytechinus pictus* (1)		
Snails				*Biomphalaria alexandrina* (3)		*Physa acuta* (3)<br>*Potamopyrgus antipodarum* (3)	*Haliotis diversicolor supertexta* (4)	*Potamopyrgus antipodarum* (2)	
Worms		*Arenicola marina* (1)<br>*Eisenia veneta* (6)<br>*Lumbriculus variegatus* (3)	*Eisenia fetida* (4)<br>*Eisenia veneta* (4)<br>*Lumbricus rubellus* (5)<br>*Lumbriculus variegatus* (2)	*Eisenia fetida* (6)		*Eisenia andrei* (4)<br>*Eisenia fetida* (4)<br>*Lumbricus terrestris* (13)	*Arenicola marina* (1)<br>*Eisenia andrei* (7)<br>*Eisenia fetida* (10)		
Yeasts						*Saccharomyces cerevisiae* (1)	*Saccharomyces cerevisiae* (1)		*Saccharomyces cerevisiae* (2)

References

1. Ivask, A.; ElBadawy, A.; Kaweeteerawat, C.; Boren, D.; Fischer, H.; Ji, Z.; Chang, C. H.; Liu, R.; Tolaymat, T.; Telesca, D.; Zink, J. I.; Cohen, Y.; Holden, P. A.; Godwin, H. A. *ACS Nano.* **2014,** *8,* 374-386.
2. Radniecki, T. S.; Stankus, D. P.; Neigh, A.; Nason, J. A.; Semprini, L. *Chemosphere.* **2011,** *85,* 43-49.
3. Calder, A. J.; Dimkpa, C. O.; McLean, J. E.; Britt, D. W.; Johnson, W.; Anderson, A. J. *Sci Total Environ.* **2012,** *429,* 215-222.
4. Engelke, M.; Koeser, J.; Hackmann, S.; Zhang, H.; Maedler, L.; Filser, J. *Environ Toxicol Chem.* **2014,** *33,* 1142-1147.
5. Ivask, A.; Bondarenko, O.; Jepihhina, N.; Kahru, A. *Anal Bioanal Chem.* **2010,** *398,* 701-716.
6. Gagne, F.; Auclair, J.; Fortier, M.; Bruneau, A.; Fournier, M.; Turcotte, P.; Pilote, M.; Gagnon, C. *J Toxicol Environ Health, A: Curr Iss.* **2013,** *76,* 767-777.
7. Lee, Y.-J.; Kim, J.; Oh, J.; Bae, S.; Lee, S.; Hong, I. S.; Kim, S.-H. *Environ Toxicol Chem.* **2012,** *31,* 155-159.
8. Jo, H. J.; Choi, J. W.; Lee, S. H.; Hong, S. W. *J Hazard Mater.* **2012,** *227,* 301-308.
9. Allen, H. J.; Impellitteri, C. A.; Macke, D. A.; Heckman, J. L.; Poynton, H. C.; Lazorchak, J. M.; Govindaswamy, S.; Roose, D. L.; Nadagouda, M. N. *Environ Toxicol Chem.* **2010,** *29,* 2742-2750.
10. Schlich, K.; Klawonn, T.; Terytze, K.; Hund-Rinke, K. *Environ Toxicol Chem.* **2013,** *32,* 181-188.
11. Kaveh, R.; Li, Y.-S.; Ranjbar, S.; Tehrani, R.; Brueck, C. L.; Van Aken, B. *Environ Sci Technol.* **2013,** *47,* 10637-10644.
12. Oukarroum, A.; Barhoumi, L.; Pirastru, L.; Dewez, D. *Environ Toxicol Chem.* **2013,** *32,* 902-907.
13. Stampoulis, D.; Sinha, S. K.; White, J. C. *Environ Sci Technol.* **2009,** *43,* 9473-9479.
14. Juganson, K.; Mortimer, M.; Ivask, A.; Kasemets, K.; Kahru, A. *Env Sci Process Impact.* **2013,** *15,* 244-250.
15. Niazi, J. H.; Sang, B.-I.; Kim, Y. S.; Gu, M. B. *Appl Biochem Biotechnol.* **2011,** *164,* 1278-1291.
16. Luna-delRisco, M.; Orupold, K.; Dubourguier, H.-C. *J Hazard Mater.* **2011,** *189,* 603-608.
17. Ko, K.-S.; Kong, I. C. *Appl Microbiol Biotechnol.* **2014,** *98,* 3295-3303.
18. Rousk, J.; Ackermann, K.; Curling, S. F.; Jones, D. L. *PLoS One.* **2012,** *7.*
19. Heinlaan, M.; Ivask, A.; Blinova, I.; Dubourguier, H.-C.; Kahru, A. *Chemosphere.* **2008,** *71,* 1308-1316.
20. Blinova, I.; Ivask, A.; Heinlaan, M.; Mortimer, M.; Kahru, A. *Environ Pollut.* **2010,** *158,* 41-47.
21. Pradhan, A.; Seena, S.; Pascoal, C.; Cassio, F. *Chemosphere.* **2012,** *89,* 1142-1150.
22. Li, M.; Zhu, L.; Lin, D. *Environ Sci Technol.* **2011,** *45,* 1977-1983.
23. Wiench, K.; Wohlleben, W.; Hisgen, V.; Radke, K.; Salinas, E.; Zok, S.; Landsiedel, R. *Chemosphere.* **2009,** *76,* 1356-1365.
24. Fairbairn, E. A.; Keller, A. A.; Maedler, L.; Zhou, D.; Pokhrel, S.; Cherr, G. N. *J Hazard Mater.* **2011,** *192,* 1565-1571.
25. Kumar, D.; Kumari, J.; Pakrashi, S.; Dalai, S.; Raichur, A. M.; Sastry, T. P.; Mandal, A. B.; Chandrasekaran, N.; Mukherjee, A. *Ecotoxicol Environ Saf.* **2014,** *108,* 152-160.
26. Scanlan, L. D.; Reed, R. B.; Loguinov, A. V.; Antczak, P.; Tagmount, A.; Aloni, S.; Nowinski, D. T.; Luong, P.; Tran, C.; Karunaratne, N.; Don, P.; Lin, X. X.; Falciani, F.; Higgins, C. P.; Ranville, J. F.; Vulpe, C. D.; Gilbert, B. *ACS Nano.* **2013,** *7,* 10681-10694.
27. Harmon, A. R.; Kennedy, A. J.; Poda, A. R.; Bednar, A. J.; Chappell, M. A.; Steevens, J. A. *Environ Toxicol Chem.* **2014,** *33,* 1783-1791.
28. Manusadzianas, L.; Caillet, C.; Fachetti, L.; Gylyte, B.; Grigutyte, R.; Jurkoniene, S.; Karitonas, R.; Sadauskas, K.; Thomas, F.; Vitkus, R.; Ferard, J.-F. *Environ Toxicol Chem.* **2012,** *31,* 108-114.
29. Zhang, D.; Hua, T.; Xiao, F.; Chen, C.; Gersberg, R. M.; Liu, Y.; Ng, W. J.; Tan, S. K. *Ecol Eng.* **2014,** *70,* 114-123.
30. Clement, L.; Hurel, C.; Marmier, N. *Chemosphere.* **2013,** *90,* 1083-1090.

31. Seitz, F.; Rosenfeldt, R. R.; Schneider, S.; Schulz, R.; Bundschuh, M. *Sci Total Environ.* **2014,** *493,* 891-897.
32. Angelstorf, J. S.; Ahlf, W.; von der Kammer, F.; Heise, S. *Environ Toxicol Chem.* **2014,** *33,* 2288-2296.
33. Manzo, S.; Rocco, A.; Carotenuto, R.; Picione, F. D. L.; Miglietta, M. L.; Rametta, G.; Di Francia, G. *Environ Sci Pollut Res.* **2011,** *18,* 756-763.
34. Ribeiro, F.; Gallego-Urrea, J. A.; Jurkschat, K.; Crossley, A.; Hassellov, M.; Taylor, C.; Soares, A. M. V. M.; Loureiro, S. *Sci Total Environ.* **2014,** *466,* 232-241.
35. Macken, A.; Byrne, H. J.; Thomas, K. V. *Ecotoxicol Environ Saf.* **2012,** *86,* 101-110.
36. Osborne, O. J.; Johnston, B. D.; Moger, J.; Balousha, M.; Lead, J. R.; Kudoh, T.; Tyler, C. R. *Nanotoxicology.* **2013,** *7,* 1315-1324.
37. Laban, G.; Nies, L. F.; Turco, R. F.; Bickham, J. W.; Sepulveda, M. S. *Ecotoxicology.* **2010,** *19,* 185-195.
38. Roh, J.-y.; Sim, S. J.; Yi, J.; Park, K.; Chung, K. H.; Ryu, D.-y.; Choi, J. *Environ Sci Technol.* **2009,** *43,* 3933-3940.
39. Mortimer, M.; Kasemets, K.; Vodovnik, M.; Marinsek-Logar, R.; Kahru, A. *Environ Sci Technol.* **2011,** *45,* 6617-6624.
40. Pang, C.; Selck, H.; Banta, G. T.; Misra, S. K.; Berhanu, D.; Dybowska, A.; Valsami-Jones, E.; Forbes, V. E. *Environ Toxicol Chem.* **2013,** *32,* 1561-1573.
41. Santo, N.; Fascio, U.; Torres, F.; Guazzoni, N.; Tremolada, P.; Bettinetti, R.; Mantecca, P.; Bacchetta, R. *Water Res.* **2014,** *53,* 339-350.
42. Yu, L.-p.; Fang, T.; Xiong, D.-w.; Zhu, W.-t.; Sima, X.-f. *J Environ Monit.* **2011,** *13,* 1975-1982.
43. Gou, N.; Gu, A. Z. *Environ Sci Technol.* **2011,** *45,* 5410-5417.
44. Fabrega, J.; Renshaw, J. C.; Lead, J. R. *Environ Sci Technol.* **2009,** *43,* 9004-9009.
45. Hu, C.; Li, M.; Wang, W.; Cui, Y.; Chen, J.; Yang, L. *Toxicol Environ Chem.* **2012,** *94,* 732-741.
46. Manier, N.; Bado-Nilles, A.; Delalain, P.; Aguerre-Chariol, O.; Pandard, P. *Environ Pollut.* **2013,** *180,* 63-70.
47. Rodea-Palomares, I.; Boltes, K.; Fernandez-Pinas, F.; Leganes, F.; Garcia-Calvo, E.; Santiago, J.; Rosal, R. *Toxicol Sci.* **2011,** *119,* 135-145.
48. Long, Z.; Ji, J.; Yang, K.; Lin, D.; Wu, F. *Environ Sci Technol.* **2012,** *46,* 8458-8466.
49. Liu, S.; Wei, L.; Hao, L.; Fang, N.; Chang, M. W.; Xu, R.; Yang, Y.; Chen, Y. *ACS Nano.* **2009,** *3,* 3891-3902.
50. Templeton, R. C.; Ferguson, P. L.; Washburn, K. M.; Scrivens, W. A.; Chandler, G. T. *Environ Sci Technol.* **2006,** *40,* 7387-7393.
51. Simon-Deckers, A.; Loo, S.; Mayne-L'Hermite, M.; Herlin-Boime, N.; Menguy, N.; Reynaud, C.; Gouget, B.; Carriere, M. *Environ Sci Technol.* **2009,** *43,* 8423-8429.
52. Xiong, D.; Fang, T.; Yu, L.; Sima, X.; Zhu, W. *Sci Total Environ.* **2011,** *409,* 1444-1452.
53. Lee, W.-M.; An, Y.-J. *Chemosphere.* **2013,** *91,* 536-544.
54. Dasari, T. P.; Hwang, H.-M. *Sci Total Environ.* **2010,** *408,* 5817-5823.
55. Nair, P. M. G.; Park, S. Y.; Choi, J. *Chemosphere.* **2013,** *92,* 592-599.
56. Zhang, H.; He, X.; Zhang, Z.; Zhang, P.; Li, Y.; Ma, Y.; Kuang, Y.; Zhao, Y.; Chai, Z. *Environ Sci Technol.* **2011,** *45,* 3725-3730.
57. Pereira, M. M.; Mouton, L.; Yepremian, C.; Coute, A.; Lo, J.; Marconcini, J. M.; Ladeira, L. O.; Raposo, N. R. B.; Brandao, H. M.; Brayner, R. *J Nanobiotechnology.* **2014,** *12.*
58. Klaper, R.; Crago, J.; Barr, J.; Arndt, D.; Setyowati, K.; Chen, J. *Environ Pollut.* **2009,** *157,* 1152-1156.
59. Hao, L.; Wang, Z.; Xing, B. *J Environ Sci-China.* **2009,** *21,* 1459-1466.
60. Kumar, A.; Pandey, A. K.; Singh, S. S.; Shanker, R.; Dhawan, A. *Free Radical Biol Med.* **2011,** *51,* 1872-1881.
61. Hu, C. W.; Li, M.; Cui, Y. B.; Li, D. S.; Chen, J.; Yang, L. Y. *Soil Biol Biochem.* **2010,** *42,* 586-591.
62. Hao, L.; Chen, L. *Ecotoxicol Environ Saf.* **2012,** *80,* 103-110.

63. Fahmy, S. R.; Abdel-Ghaffar, F.; Bakry, F. A.; Sayed, D. A. *Arch Environ Contam Toxicol.* **2014,** *67,* 192-202.
64. Park, S.-y.; Choi, J. *Environ Eng Res.* **2010,** *15,* 23-27.
65. Nair, P. M. G.; Park, S. Y.; Lee, S.-W.; Choi, J. *Aquat Toxicol.* **2011,** *101,* 31-37.
66. Ghosh, M.; Manivannan, J.; Sinha, S.; Chakraborty, A.; Mallick, S. K.; Bandyopadhyay, M.; Mukherjee, A. *Mutat Res-Genet Toxicol Environ Mutag.* **2012,** *749,* 60-69.
67. Schwab, F.; Bucheli, T. D.; Lukhele, L. P.; Magrez, A.; Nowack, B.; Sigg, L.; Knauer, K. *Environ Sci Technol.* **2011,** *45,* 6136-6144.
68. Gaiser, B. K.; Biswas, A.; Rosenkranz, P.; Jepson, M. A.; Lead, J. R.; Stone, V.; Tyler, C. R.; Fernandes, T. F. *J Environ Monit.* **2011,** *13,* 1227-1235.
69. Artells, E.; Issartel, J.; Auffan, M.; Borschneck, D.; Thill, A.; Tella, M.; Brousset, L.; Rose, J.; Bottero, J.-Y.; Thiery, A. *PLoS One.* **2013,** *8*.
70. Dabrunz, A.; Duester, L.; Prasse, C.; Seitz, F.; Rosenfeldt, R.; Schilde, C.; Schaumann, G. E.; Schulz, R. *PLoS One.* **2011,** *6*.
71. Farkas, J.; Christian, P.; Urrea, J. A. G.; Roos, N.; Hassellov, M.; Tollefsen, K. E.; Thomas, K. V. *Aquat Toxicol.* **2010,** *96,* 44-52.
72. Fabrega, J.; Zhang, R.; Renshaw, J. C.; Liu, W.-T.; Lead, J. R. *Chemosphere.* **2011,** *85,* 961-966.
73. Resano, M.; Lapena, A. C.; Belarra, M. A. *Anal Methods.* **2013,** *5,* 1130-1139.
74. Musante, C.; White, J. C. *Environ Toxicol.* **2012,** *27,* 510-517.
75. Bourdiol, F.; Mouchet, F.; Perrault, A.; Fourquaux, I.; Datas, L.; Gancet, C.; Boutonnet, J.-C.; Pinelli, E.; Gauthier, L.; Flahaut, E. *Carbon.* **2013,** *54,* 175-191.
76. Hanna, S. K.; Miller, R. J.; Lenihan, H. S. *J Hazard Mater.* **2014,** *279,* 32-37.
77. Petersen, E. J.; Akkanen, J.; Kukkonen, J. V. K.; Weber, W. J., Jr. *Environ Sci Technol.* **2009,** *43,* 2969-2975.
78. Petersen, E. J.; Pinto, R. A.; Mai, D. J.; Landrum, P. F.; Weber, W. J., Jr. *Environ Sci Technol.* **2011,** *45,* 1133-1138.
79. Dai, L.; Syberg, K.; Banta, G. T.; Selck, H.; Forbes, V. E. *ACS Sustain Chem Eng.* **2013,** *1,* 760-767.
80. Hu, J.; Wang, D.; Wang, J.; Wang, J. *Environ Pollut.* **2012,** *162,* 216-222.
81. Chen, P.-J.; Tan, S.-W.; Wu, W.-L. *Environ Sci Technol.* **2012,** *46,* 8431-8439.
82. Pakarinen, K.; Petersen, E. J.; Leppanen, M. T.; Akkanen, J.; Kukkonen, J. V. K. *Environ Pollut.* **2011,** *159,* 3750-3756.
83. Pakarinen, K.; Petersen, E. J.; Alvila, L.; Waissi-Leinonen, G. C.; Akkanen, J.; Leppanen, M. T.; Kukkonen, J. V. K. *Environ Toxicol Chem.* **2013,** *32,* 1224-1232.
84. Zhu, X.; Zhu, L.; Chen, Y.; Tian, S. *J Nanopart Res.* **2009,** *11,* 67-75.
85. Jovanovic, B.; Guzman, H. M. *Environ Toxicol Chem.* **2014,** *33,* 1346-1353.
86. Zhu, X.; Chang, Y.; Chen, Y. *Chemosphere.* **2010,** *78,* 209-215.
87. Sun, H.; Zhang, X.; Niu, Q.; Chen, Y.; Crittenden, J. C. *Water Air Soil Poll.* **2007,** *178,* 245-254.
88. Zhang, X.; Sun, H.; Zhang, Z.; Niu, Q.; Chen, Y.; Crittenden, J. C. *Chemosphere.* **2007,** *67,* 160-166.
89. Sun, H.; Zhang, X.; Zhang, Z.; Chen, Y.; Crittenden, J. C. *Environ Pollut.* **2009,** *157,* 1165-1170.
90. Zhu, X.; Wang, J.; Zhang, X.; Chang, Y.; Chen, Y. *Chemosphere.* **2010,** *79,* 928-933.
91. Larue, C.; Castillo-Michel, H.; Sobanska, S.; Trcera, N.; Sorieul, S.; Cecillon, L.; Ouerdane, L.; Legros, S.; Sarret, G. *J Hazard Mater.* **2014,** *273,* 17-26.
92. Larue, C.; Laurette, J.; Herlin-Boime, N.; Khodja, H.; Fayard, B.; Flank, A.-M.; Brisset, F.; Carriere, M. *Sci Total Environ.* **2012,** *431,* 197-208.
93. Asghari, S.; Johari, S. A.; Lee, J. H.; Kim, Y. S.; Jeon, Y. B.; Choi, H. J.; Moon, M. C.; Yu, I. J. *J Nanobiotechnology.* **2012,** *10.*

94. Schwabe, F.; Schulin, R.; Limbach, L. K.; Stark, W.; Buerge, D.; Nowack, B. *Chemosphere.* **2013,** *91*, 512-520.
95. Mwangi, J. N.; Wang, N.; Ingersoll, C. G.; Hardesty, D. K.; Brunson, E. L.; Li, H.; Deng, B. *Environ Toxicol Chem.* **2012,** *31*, 1823-1830.
96. Martinez, D. S. T.; Faria, A. F.; Berni, E.; Souza Filho, A. G.; Almeida, G.; Caloto-Oliveira, A.; Grossman, M. J.; Durrant, L. R.; Umbuzeiro, G. A.; Alves, O. L. *Process Biochem.* **2014,** *49*, 1162-1168.
97. Roberts, A. P.; Mount, A. S.; Seda, B.; Souther, J.; Qiao, R.; Lin, S.; Ke, P. C.; Rao, A. M.; Klaine, S. J. *Environ Sci Technol.* **2007,** *41*, 3025-3029.
98. Lin, S.; Reppert, J.; Hu, Q.; Hudson, J. S.; Reid, M. L.; Ratnikova, T. A.; Rao, A. M.; Luo, H.; Ke, P. C. *Small.* **2009,** *5*, 1128-1132.
99. Planchon, M.; Ferrari, R.; Guyot, F.; Gelabert, A.; Menguy, N.; Chaneac, C.; Thill, A.; Benedetti, M. F.; Spalla, O. *Colloids Surf B Biointerfaces.* **2013,** *102*, 158-164.
100. Li, L.; Sillanpaa, M.; Tuominen, M.; Lounatmaa, K.; Schultz, E. *Ecotoxicol Environ Saf.* **2013,** *88*, 89-94.
101. Jarvis, T. A.; Miller, R. J.; Lenihan, H. S.; Bielmyer, G. K. *Environ Toxicol Chem.* **2013,** *32*, 1264-1269.
102. Lin, D.; Xing, B. *Environ Sci Technol.* **2008,** *42*, 5580-5585.
103. Mouchet, F.; Landois, P.; Puech, P.; Pinelli, E.; Flahaut, E.; Gauthier, L. *Nanomedicine.* **2010,** *5*, 963-974.
104. Kennedy, A. J.; Gunter, J. C.; Chappell, M. A.; Goss, J. D.; Hull, M. S.; Kirgan, R. A.; Steevens, J. A. *Environ Toxicol Chem.* **2009,** *28*, 1930-1938.
105. Kowk, K. W. H.; Leung, K. M. Y.; Flahaut, E.; Cheng, J.; Cheng, S. H. *Nanomedicine.* **2010,** *5*, 951-961.
106. Chan, T. S. Y.; Nasser, F.; St-Denis, C. H.; Mandal, H. S.; Ghafari, P.; Hadjout-Rabi, N.; Bols, N. C.; Tang, X. *Nanotoxicology*. **2013,** *7*, 251-258.
107. Strigul, N.; Vaccari, L.; Galdun, C.; Wazne, M.; Liu, X.; Christodoulatos, C.; Jasinkiewicz, K. *Desalination.* **2009,** *248*, 771-782.
108. Kim, K. T.; Klaine, S. J.; Cho, J.; Kim, S.-H.; Kim, S. D. *Sci Total Environ.* **2010,** *408*, 2268-2272.
109. Galloway, T.; Lewis, C.; Dolciotti, I.; Johnston, B. D.; Moger, J.; Regoli, F. *Environ Pollut.* **2010,** *158*, 1748-1755.
110. Kumar, A.; Pandey, A. K.; Singh, S. S.; Shanker, R.; Dhawan, A. *Cytometry Part A.* **2011,** *79A*, 707-712.
111. Dalai, S.; Pakrashi, S.; Chandrasekaran, N.; Mukherjee, A. *PLoS One.* **2013,** *8*.
